# PKA phosphorylation underlies functional recruitment of sarcolemmal SK2 channels in ventricular myocytes from hypertrophic hearts

**DOI:** 10.1113/JP277618

**Published:** 2019-03-20

**Authors:** Shanna Hamilton, Iuliia Polina, Radmila Terentyeva, Peter Bronk, Tae Yun Kim, Karim Roder, Richard T. Clements, Gideon Koren, Bum‐Rak Choi, Dmitry Terentyev

**Affiliations:** ^1^ Department of Medicine The Warren Alpert Medical School of Brown University Rhode Island Hospital Cardiovascular Research Center Providence RI USA; ^2^ Dorothy M. Davis Heart and Lung Research Institute College of Medicine The Ohio State University Columbus OH USA; ^3^ Department of Physiology and Cell Biology College of Medicine The Ohio State University Columbus OH USA; ^4^ Medical University of South Carolina Department of Medicine Division of Nephrology Charleston SC USA; ^5^ Department of Surgery The Warren Alpert Medical School of Brown University Rhode Island Hospital Cardiovascular Research Center Providence RI USA; ^6^ Vascular Research Laboratory Providence Veterans Affairs Medical Center Providence RI USA

**Keywords:** Small‐conductance Ca^2+^‐activated K^+^ channels, Ventricular arrhythmia, PKA, Calcium transients, Cardiac electrophysiology

## Abstract

**Key points:**

- Small‐conductance Ca^2+^‐activated K^+^ (SK) channels expressed in ventricular myocytes are dormant in health, yet become functional in cardiac disease.
- SK channels are voltage independent and their gating is controlled by intracellular [Ca^2+^] in a biphasic manner. Submicromolar [Ca^2+^] activates the channel via constitutively‐bound calmodulin, whereas higher [Ca^2+^] exerts inhibitory effect during depolarization.
- Using a rat model of cardiac hypertrophy induced by thoracic aortic banding, we found that functional upregulation of SK2 channels in hypertrophic rat ventricular cardiomyocytes is driven by protein kinase A (PKA) phosphorylation. Using site‐directed mutagenesis, we identified serine‐465 as the site conferring PKA‐dependent effects on SK2 channel function.
- PKA phosphorylation attenuates *I*_SK_ rectification by reducing the Ca^2+^/voltage‐dependent inhibition of SK channels without changing their sensitivity to activating submicromolar [Ca^2+^]_i_.
- This mechanism underlies the functional recruitment of SK channels not only in cardiac disease, but also in normal physiology, contributing to repolarization under conditions of enhanced adrenergic drive.

**Abstract:**

Small‐conductance Ca^2+^‐activated K^+^ (SK) channels expressed in ventricular myocytes (VMs) are dormant in health, yet become functional in cardiac disease. We aimed to test the hypothesis that post‐translational modification of SK channels under conditions accompanied by enhanced adrenergic drive plays a central role in disease‐related activation of the channels. We investigated this phenomenon using a rat model of hypertrophy induced by thoracic aortic banding (TAB). Western blot analysis using anti‐pan‐serine/threonine antibodies demonstrated enhanced phosphorylation of immunoprecipitated SK2 channels in VMs from TAB rats *vs*. Shams, which was reversible by incubation of the VMs with PKA inhibitor H89 (1 μmol L^–1^). Patch clamped VMs under basal conditions from TABs but not Shams exhibited outward current sensitive to the specific SK inhibitor apamin (100 nmol L^–1^), which was eliminated by inhibition of PKA (1 μmol L^–1^). Beta‐adrenergic stimulation (isoproterenol, 100 nmol L^–1^) evoked *I*_SK_ in VMs from Shams, resulting in shortening of action potentials in VMs and *ex vivo* optically mapped Sham hearts. Using adenoviral gene transfer, wild‐type and mutant SK2 channels were overexpressed in adult rat VMs, revealing serine‐465 as the site that elicits PKA‐dependent phosphorylation effects on SK2 channel function. Concurrent confocal Ca^2+^ imaging experiments established that PKA phosphorylation lessens rectification of *I*_SK_ via reduction Ca^2+^/voltage‐dependent inhibition of the channels at high [Ca^2+^] without affecting their sensitivity to activation by Ca^2+^ in the submicromolar range. In conclusion, upregulation of SK channels in diseased VMs is mediated by hyperadrenergic drive in cardiac hypertrophy, with functional effects on the channel conferred by PKA‐dependent phosphorylation at serine‐465.

## Key points

- Small‐conductance Ca^2+^‐activated K^+^ (SK) channels expressed in ventricular myocytes are dormant in health, yet become functional in cardiac disease.
- SK channels are voltage independent and their gating is controlled by intracellular [Ca^2+^] in a biphasic manner. Submicromolar [Ca^2+^] activates the channel via constitutively‐bound calmodulin, whereas higher [Ca^2+^] exerts inhibitory effect during depolarization.
- Using a rat model of cardiac hypertrophy induced by thoracic aortic banding, we found that functional upregulation of SK2 channels in hypertrophic rat ventricular cardiomyocytes is driven by protein kinase A (PKA) phosphorylation. Using site‐directed mutagenesis, we identified serine‐465 as the site conferring PKA‐dependent effects on SK2 channel function.
- PKA phosphorylation attenuates *I*_SK_ rectification by reducing the Ca^2+^/voltage‐dependent inhibition of SK channels without changing their sensitivity to activating submicromolar [Ca^2+^]_i_.
- This mechanism underlies the functional recruitment of SK channels not only in cardiac disease, but also in normal physiology, contributing to repolarization under conditions of enhanced adrenergic drive.

## Introduction

Sudden cardiac death as a result of ventricular tachyarrhythmias remains a major cause of mortality worldwide (Benjamin *et al*. 2018). Enhanced triggered activity for arrhythmia under conditions such as heart failure at the cellular level has been largely ascribed to reduced repolarizing K^+^ currents, increased L‐type Ca^2+^ current (*I*_Ca_) and Na^+^/Ca^2+^ exchanger (NCX1)‐mediated depolarization, caused by untimely releases of sarcoplasmic reticulum (SR) Ca^2+^ by hyperactive ryanodine receptors (RyR2s) (Pogwizd & Bers, 2004; Qu & Weiss, 2006; Zima *et al*. 2014). Recently, small conductance Ca^2+^‐activated K^+^ (SK) channels have emerged as a promising therapeutic target because of their ability to offset depolarizing force of *I*_Ca_ and *I*_NCX_ and mitigate disease‐associated loss of repolarization reserve (Chang *et al*. 2015a; Clements *et al*. 2015; Terentyev *et al*. 2014). However, the exact role of SK channels in arrhythmogenesis and the mechanisms that regulate their function in the heart remain poorly understood.

All three SK channel isoforms (SK1‐3) encoded by the genes *KCNN1*, *KCNN2* and *KCNN3* are detected in cardiac tissue (Skibsbye *et al*. 2014; Tuteja *et al*. 2005; Xu *et al*. 2003). Our previous report demonstrated that two of these isoforms are present in sarcolemma of rat ventricular myocytes (VMs): SK2 and SK3 (Kim *et al*. 2017). SK channels exist as heterotetrameric multicomplex proteins with six transmembrane domains and they display small single channel conductances of ∼10–20 pS (Adelman *et al*. 2012; Tuteja *et al*. 2010). Because SK channels lack a classical voltage sensor, their gating is controlled primarily by intracellular [Ca^2+^] ([Ca^2+^]_i_) in a biphasic manner (Maylie *et al*. 2003; Soh & Park, 2002; Xia *et al*. 1998). Channel activation is conferred by calmodulin (CaM) constitutively bound to the C‐terminus, with an EC_50_ of ∼0.3‐1 μmol L^–1^ (Li N *et al*. 2009; Li W *et al*. 2009; Schumacher *et al*. 2001; Schumacher *et al*. 2004; Xia *et al*. 1998). In addition, higher [Ca^2+^]_i_ has been reported to inhibit SK channels in a voltage‐dependent manner (Soh & Park, 2002), providing the basis for rectification of *I*_SK_.

Unlike in atria (Diness *et al*. 2011; Li N *et al*. 2009; Skibsbye *et al*. 2014), SK channels in VMs are dormant in health and become active in cardiac disease both in animal models and human patients (Chang *et al*. 2013b; Chua *et al*. 2011; Clements *et al*. 2015; Bonilla *et al*. 2014; Lee *et al*. 2013; Mahida, 2014; Ni *et al*. 2013). Functional recruitment of plasmalemmal SK channels can occur very rapidly. For example, contribution of SK channels to repolarization became obvious 10 min after the induction of acute myocardial infraction or 30 min of ischaemia in rat hearts (Gui *et al*. 2012; Tenma *et al*. 2018). Several hypotheses were proposed to explain this phenomenon including: (i) increased expression levels (Chang *et al*. 2013a; Ni *et al*. 2013); (ii) increased activity as a result of CaMKII phosphorylation (Mizukami *et al*. 2015; Tenma *et al*. 2018); and (iii) an increase in sensitivity to activating Ca^2+^ as a result of dephosphorylation of SK‐bound CaM given changes in the activities of SK‐associated protein casein kinase 2 (CK2) and protein phosphatase 2A (PP2A) (Allen *et al*. 2007; Bildl *et al*. 2004; Yang *et al*. 2015; Zhang *et al*. 2014). We previously reported that functional upregulation of plasmalemmal SK channels in VMs from hypertrophic rat hearts was not paralleled by increased expression levels (Kim *et al*. 2017), suggesting that post‐translational modifications may instead underlie enhanced channel activity.

Phosphoproteomic studies revealed that SK channels can be directly phosphorylated by protein kinase A (PKA) at N‐terminal serine‐136, within the CaM‐binding domain at serine‐465, and at C‐terminal serine‐568 to serine‐570 (for rat SK2) (Blom *et al*. 1999; Ren *et al*. 2006). Interestingly, recent studies suggest that β‐adrenergic stimulation evokes apamin‐sensitive repolarizing current in ventricles of optically mapped *ex vivo* rat and rabbit hearts. (Chen *et al*. 2018; Kamada *et al*. 2018). By contrast, earlier experiments using heterologous systems suggest that PKA phosphorylation negatively affects SK function either by reducing channel activity in HEK cells (SK3) (Clarysse *et al*. 2014) or by interfering with surface localization of SK2 in COS7 cells (Ren *et al*. 2006).

To gain insight into the mechanisms of upregulation of SK channels in VMs from diseased hearts and to determine the role of PKA in this process in particular, we used a clinically relevant rat model of cardiac arrhythmia with hypertrophy induced by thoracic aortic banding (TAB) (Kim *et al*. 2017; del Monte *et al*. 2002). Using *ex vivo* optical mapping and single cell electrophysiology in conjunction with confocal Ca^2+^ imaging, we found that PKA‐dependent phosphorylation is the major determinant of functional upregulation of the channels via attenuation of voltage‐dependent inhibition by [Ca^2+^]_i_. Furthermore, using cultured rat VMs overexpressing wild‐type (WT) and mutant SK2 channels, we have identified the site within the channel responsible for functional upregulation driven by PKA‐mediated phosphorylation, namely serine‐465.

## Methods

### Ethical approval

All procedures involving animals were approved by The Rhode Island Hospital Institutional Animal Care and Use Committee and conformed with the Guide for the Care and Use of Laboratory Animals published by the US National Institutes of Health (NIH Publication No. 85‐23, revised 2011) and the policies and regulations set out in the editorial in *The Journal of Physiology and Experimental Physiology* by Grundy (2015). During procedures, all steps were taken to minimize animals pain and suffering.

Male Sham and TAB Sprague–Dawley rats (RGD catalogue no. 10395233, RRID:RGD_10395233) were purchased from Charles River Laboratories (Wilmington, MA, USA). Animals were shipped 5–7 days after surgery and acclimatized for 3–4 weeks in the Rhode Island Hospital animal facility. Experiments were performed 4–5 weeks after aortic banding procedure. Animals were fed *ad libitum*. In total, 29 Sham rats and 30 TAB rats were used for the present study.

### *In vivo* cardiac function

Male Sham and TAB Sprague–Dawley rats were sedated with continuous isoflurane (1–3%) via induction chamber and nose cone and then the chest was shaved. Transthoracic M‐mode and two‐dimensional echocardiography was performed on a Vevo® 2100 Imaging System (Fujifilm VisualSonics, Inc., Toronto, ON, Canada). The analysis included recording the dimensions of the left ventricle, as well as the total heart weight/body weight ratio. The entire procedure took 15–30 min and resulted in no pain to the animal. During the procedure, animals were closely monitored for any signs of distress, temperature and heart rate changes via an ECG. Rats were then monitored during recovery from anaesthesia for 1–2 h to ensure normal movement and activity before being returned to normal housing.

### Myocyte isolation and cell culture

Whole hearts and VMs were isolated from male Sham and TAB Sprague–Dawley rats. Rats were injected with 120 mg kg^–1^ pentobarbital i.p. as a terminal procedure. The heart was removed from the rats via bilateral thoracotomy, mounted on optical mapping set up or a Langendorff apparatus and retrogradely perfused with Tyrode solution containing collagenase II (Worthington Biochemical Corp. Lakewood, NJ, USA) at 37°C. VMs were isolated as described previously (Terentyev *et al*. 2014), before plating onto laminin‐coated coverslips. VMs were used within 8 h of isolation. For experiments with rat SK2 (rSK2) channel overexpression, adult rat VMs were isolated from 9–12‐week old Sprague–Dawley male rats (RGD catalogue no. 70508; RRID:RGD_70508) from Harlan Laboratories (Indianapolis, IN, USA), as described previously (Terentyev *et al*. 2014). Myocytes were plated in 24‐well plates on laminin‐coated glass coverslips, cultured in serum‐free medium 199 (Thermo Fisher Scientific, Waltham, MA, USA), supplemented with 25 mmol L^–1^ NaHCO_3_, 10 mmol L^–1^ Hepes, 5 mmol L^–1^ creatine, 5 mmol L^–1^ taurine, 10 U mL^–1^ penicillin, 10 μg mL^–1^ streptomycin and 10 μg mL^–1^ gentamycin (pH 7.3). Unattached cells were removed after 1 h and the remaining VMs were infected with adenoviruses at a multiplicity of infection of 10 for SK channel and dominant‐negative mutant phospholamban virus (dnPLB) constructs. Myocytes were cultured at 37°C in 95% air/5% CO_2_ for 36–48 h before the analysis.

### Construction of WT and mutant SK2 adenoviruses

Adenovirus carrying recombinant WT rSK2 sequence was constructed as described previously (Terentyev *et al*. 2014; Kim *et al*. 2017), utilizing the ViraPower Gateway expression system (Thermo Fisher Scientific). Briefly, the coding region of rat SK2 sequence was cloned into pENTR™ 1A vector, and then recombined into pAd/CMV/V5‐DEST™ with the LR recombination reaction. Sequence‐verified plasmid was digested with restriction enzyme *Pac*I, before transfection into HEK293A cells (RRID:CVCL_6910) using Lipofectamine™ 2000 (Invitrogen, Carlsbad, CA, USA). Titre of amplified adenoviral stocks was determined using the Adeno‐X qPCR Titration Kit (Takara Bio USA, Inc., Mountain View, CA, USA). To introduce S136D into rSK2 construct, we applied site‐directed mutagenesis using the Quik Change Site‐Directed Mutagenesis Kit (Agilent Technologies Inc., Santa Clara, CA, USA). To introduce phosphomimetic mutation S465D and S465A into rat SK2 construct, we applied site‐directed mutagenesis using the Q5^®^ Site‐Directed Mutagenesis Kit (New England Biolabs Inc., Ipswich, MA, USA) in accordance with the manufacturer's instructions. dnPLB with K3E/R14E mutations was used to enhance SERCa2a function and restore Ca^2+^ transient amplitude (a kind gift from Dr M. Ziolo, The Ohio State University, Columbus, OH, USA) (Ziolo *et al*. 2005). A virus with non‐coding sequence following CMV was used as a control.

### Western blotting and immunoprecipitation

The antibodies used were SK2 (Sigma, St Louis, MO, USA; catalogue no. SAB2501396, Lot#9678P1, RRID:AB_10961767; dilution 1:2000; Alomone Labs, Jerusalem, Israel; catalogue no. APC‐028, Lot#APC028AN1702, RRID:AB_2040126; dilution 1:1000), GAPDH (Abcam, Cambridge, MA, USA; catalogue no. ab8245, Lot#GR232949‐15, RRID:AB_2107448; dilution 1:5000), calmodulin (CaM) (Abcam; catalogue no. ab45689, Lot#gr291267‐5, RRID:AB_1946552; dilution 1:1000), phospho‐calmodulin (phosphoCaM) (Abcam; catalogue no. ab61001, Lot#GR10051348, RRID:AB_942205; dilution 1:1000), casein kinase 2a E‐2 (CK2) (Santa Cruz Biotechnology, Dallas, TX, USA; catalogue no. sc‐365787, Lot#E2411, RRID:AB_10844012; dilution 1:1000), protein phosphatase 2A C subunit (PP2A‐C) (Millipore, Burlington, MA, USA; catalogue no. 05‐421, Lot#JBC1856024, RRID:AB_309726; dilution 1:1000), Ca^2+^/calmodulin‐dependent kinase II (CaMKII) (Thermo Fisher Scientific; catalogue no. MA1‐048, Lot#CVP107, RRID:AB_325403; dilution 1:1000), phospho‐Ca^2+^/calmodulin‐dependent kinase II (phospho‐CaMKII) (Thermo Fisher Scientific; catalogue no. PA1‐4614, Lot#196‐120, RRID:AB_2259386; dilution 1:1000 dilution), phospho‐PKA substrate (Cell Signaling Technology, Danvers, MA, USA; catalogue no. 9621, Lot#20, RRID:AB_330304; dilution 1:2000), phospho‐serine/threonine (Abcam; catalogue no. ab17464, Lot#GR3209832‐1, RRID:AB_443891; dilution 1:1000) and phospholamban [PLB; (2D12)] (a kind gift from Dr Z. Chen, Indiana University School of Medicine, Indianapolis, IN, USA; Chen *et al*. 2007; dilution 1:1000). Custom polyclonal rat phospho‐S465‐SK2 site‐specific antibodies were generated by YenZym Antibodies, LLC (San Francisco, CA, USA) by immunization of rabbits with the peptide LRpSVKMEQRKLNDQC. Antibodies were affinity‐purified by enzyme‐linked immunosorbent assay.

Freshly isolated or cultured rat VMs were lysed in lysis buffer from Cell Signaling (catalogue no. 9803S), supplemented with phosphatase (Calbiochem, San Diego, CA, USA; catalogue no. 524625) and protease inhibitor cocktails (Sigma; catalogue no. P8340) as described previously (Terentyev *et al*. 2014). Samples (20–30 μg of proteins) were resolved on a 4–20% gel via SDS‐PAGE, transferred onto nitrocellulose membranes, and probed with antibodies specific for these proteins and subsequently probed with a goat anti‐mouse secondary (Promega, Madison, WI, USA; catalogue no. W4021, Lot#0000292575, RRID:AB_430834; dilution 1:10,000 ), goat anti‐rabbit secondary (Promega; catalogue no. W4011, Lot#0000292577, RRID:AB_430833; dilution 1:10,000) or donkey anti‐goat secondary antibodies (Promega; catalogue no. V805A, Lot#0000265854, RRID:AB_430838; dilution 1:10,000). Blots were developed with ECL (Bio‐Rad Laboratories, Hercules, CA, USA; catalogue no. 1705061) and quantified and analysed using ImageJ (NIH, Bethesda, MD, USA; RRID: SCR_003070) and Origin, version 8 (OriginLab Corp., Northampton, MA, USA; RRID:SCR_014212).

For immunoprecipitation, freshly isolated rat VMs were lysed using cell lysis buffer from Cell Signaling (catalogue no. 9803S), supplemented with phosphatase (Calbiochem; catalogue no. 524625) and protease inhibitor cocktails (Sigma; catalogue no. P8340). A 2 h long immunoprecipitation of SK2 was performed at 4°C using a Catch and Release v2.0 Kit (Millipore; catalogue no. 17‐500) in accordance with the manufacturer instructions using anti‐SK2 antibody (Sigma; catalogue no. SAB2501396, Lot#9678P1, RRID:AB_10961767; 5 μg of antibody) and a negative control antibody comprising normal mouse IgG (Santa Cruz Biotechnology; catalogue no. sc‐2025, Lot#J2015, RRID:AB_737182; 5 μg of antibody). Samples were analysed by immunoblotting.

For the assessment of native protein complexes using blue native polyacrylamide gel electrophoresis (BN‐PAGE), we used freshly isolated VMs suspended in buffer containing 225 mmol L^–1^ mannitol, 70 mmol L^–1^ sucrose, 10 mmol L^–1^ Hepes and 1 mmol L^–1^ EGTA (pH 7.4). The whole‐cell suspension was placed in a pre‐cooled 5 mL Wheaton™ Potter‐Elveheim Tissue Grinder (Fisher Scientific, Hampton, NH, USA; catalogue no. 22‐290067). The cells were homogenized and homogenate was centrifuged at 700 *g* for 10 min. The pellet consisting of nuclei and cell debris was discarded and the supernatant was then centrifuged at 17,000 *g* for 15 min. The brown pellet was considered as mitochondrial fraction and discarded. The supernatant was used to precipitate membrane fraction at 100,000 *g* for 1 h. Samples were then solubilized using the NativePAGE™ Sample Prep Kit (Invitrogen; catalogue no. BN2008, Lot#1619815) in accordance with the manufacturer's instructions. Processed samples were resolved on NativePAGE™ 4–16% Bis‐Tris Protein Gels, 1.0 mm, 15‐well (Invitrogen; catalogue no. BN1004BOX, Lot#18061260) by SDS‐PAGE at 150 V for 1 h and 250 V for 1.5 h. Samples were transferred onto nitrocellulose membranes before being probed with antibodies, as described above. The primary antibodies used were SK2 (Sigma; catalogue no. SAB2501396, Lot#9678P1, RRID:AB_10961767; dilution 1:2000), Cav1.2α1c L‐type Ca^2+^ channel (LTCC) subunit (Alomone Labs; catalogue no. ACC‐003, Lot#ACC013AN0502, RRID:AB_2039771; dilution 1:1000), Na^+^/K^+^‐ATPase (NKA; Abcam; catalogue no. ab76020, Lot#GR3184452‐8, RRID:AB_1310695; dilution 1:1000). The secondary antibodies used are described above.

In experiments utilizing site‐specific phospho‐SK2‐S465 antibodies (dilution 1:500), maximum phosphorylation for normalization was achieved by incubation of TAB and Sham VMs with β‐adrenergic agonist isoproterenol (ISO) (1 μmol L^–1^) and phosphatase inhibitor (1 μmol L^–1^) calyculin A for 15 min.

### Proximity ligation assay and immunofluorescence

The Duolink® proximity ligation assay (PLA) (Duolink® In Situ Detection Reagents Orange, Sigma; catalogue no. DUO92007, Lot#SLBV3905) allows for the detection of proteins that are colocalized <40 nm of each other. Oligonucleotide labelled secondary antibodies or PLA probes generate signal only when bound in close proximity to two primary antibodies that have bound to the sample in close proximity.

Freshly isolated VMs were plated on laminin‐coated coverslips and prepared for the PLA and immunofluorescence by fixing with 4% paraformaldehyde and permeabilized with 0.2% Triton X‐100/PBS (pH 7.2) containing 1% BSA. The PLA probe protocol was followed in accordance with the manufacturer's instructions. Briefly, samples were blocked and washed before incubation with primary antibodies for 1 h at room temperature. Primary antibodies used were SK2 (Sigma; catalogue no. SAB2501396, Lot#9678P1, RRID:AB_10961767; dilution 1:2000) and Cav1.2α1c subunit (Alomone Labs; catalogue no. ACC‐003, Lot#ACC013AN0502, RRID:AB_2039771; dilution 1:1000) or RyR2 (Thermo Fisher Scientific; catalogue no. MA3‐916, Lot#SD241387, RRID:AB_2183054; dilution 1:1000). Next, samples were washed and PLA probes added and for ligation they were incubated for 1 h at 37 °C. PLA probes used were Duolink® In Situ PLA® Probe Anti‐Goat PLUS (Sigma; catalogue no. DUO92003, Lot# SLBW7564; dilution in accordance with the manufacturer's instructions) and Duolink® In Situ PLA® Probe Anti‐Rabbit MINUS (Sigma; catalogue no. DUO92005, Lot#SLBZ4516; dilution in accordance with the manufacturer's instructions). PLA probes were then amplified in accordance with the manufacturer's instructions using a DNA ligase at 37 °C, and then samples were immediately processed for imaging. Samples were also probed for RyR2. Primary antibody used was RyR2 (Thermo Fisher Scientific; catalogue no. MA3‐916, Lot#SD241387, RRID:AB_2183054; dilution 1:1000) or anti‐calsequestrin (CSQ) (Affinity Bioreagents, Golden, CO, USA; catalogue no. PA1‐913, Lot#387‐112, RRID:AB_2071461; dilution 1:5000). Secondary antibody used was rabbit anti‐mouse IgG (H+L) cross‐adsorbed secondary antibody, Alexa Fluor 488 (Thermo Fisher Scientific; catalogue no. A‐11059, Lot#1567256, RRID:AB_2534106; dilution 1:1000).

Images were acquired using a SP5 II confocal system (Leica Microsystems, Wetzlar, Germany) equipped with a 63 × 1.4 numerical aperture oil objective using the 488 nm line of the argon ion laser and the 546 nm line of the HeNe laser for excitation. Emitted fluorescence was collected at the wavelengths 500–530 nm and 560–660 nm. The signal from each detected pair of PLA probes was visualized as a fluorescent red spot, and was quantified as PLA puncta μm^–2^ in ImageJ (NIH; RRID: SCR_003070). Manders overlap coefficients were calculated to assess the level of colocalization between SK2‐LTCC PLA puncta and RyR2 in ImageJ (NIH; RRID: SCR_003070), with an M1 coefficient of 1.0 indicating complete colocalization of red to green images and an M2 coefficient of 1.0 indicating complete colocalization of green to red images.

### Whole‐cell patch clamp of VMs

Whole‐cell patch clamp recordings of currents and membrane potential were carried out using an Axopatch 200B amplifier (Molecular Devices, Sunnyvale, CA, USA) filtered at 2 kHz and digitized at a sampling rate of 5 kHz as described previously (Terentyev *et al*. 2014). To record SK channel currents from rat VMs, depolarizing voltage steps from a holding potential of –40 mV at 10 mV intervals were applied at 2 s intervals under voltage clamp at room temperature. For recordings from rat VMs overexpression SK2, voltage steps were from a holding potential of –45 mV. Action potentials (APs) were elicited by short current pulses applied at 1.2× threshold under current clamp. Bath solution (pH 7.3) contained 140 mmol L^–1^ NaCl, 5.4 mmol L^–1^ KCl, 1 mmol L^–1^ MgCl_2_, 1 mmol L^–1^ CaCl_2_, 10 mmol L^–1^ Hepes and 5.6 mmol L^–1^ glucose. Recording electrodes were 2–4 MΩ containing nominally Ca^2+^ free pipette solution (pH 7.2): 90 mmol L^–1^ K‐aspartate, 50 mmol L^–1^ KCl, 5 mmol L^–1^ Mg‐ATP, 5 mmol L^–1^ NaCl, 1 mmol L^–1^ MgCl_2_, 0.1 mmol L^–1^ Tris‐GTP, 10 mmol L^–1^ Hepes and 0.1 mmol L^–1^ Rhod‐2 K^+^‐salt (Thermo Fisher Scientific). Free [Mg^2+^] was 1.37 mmol L^–1^ (Maxchelator) (Bers *et al*. 2010). Usually, the series resistance was compensated 40–60%. Apamin (APA), a selective SK1, 2 and 3 polypeptide inhibitor (IC_50 _< 10 nmol L^–1^; Alomone Labs) was used to identify SK currents. UCL‐1684 is a non‐peptidic voltage independent blocker of SK channels (IC_50 _< 10 nmol L^–1^) (Hosseini *et al*. 2001; Rosa *et al*. 1998). For β‐adrenergic stimulation, VMs were treated with ISO (100 nmol L^–1^). To inhibit PKA, synthetic peptide PKA Inhibitor 14–22 Amide (PKI) (Calbiochem; catalogue no. 476485) was added to pipette solution (1 μmol L^–1^).

### Confocal imaging and estimation of [Ca^2+^]_i_

During whole‐cell voltage clamp experiments, intracellular Ca^2+^ imaging was simultaneously performed at room temperature using a SP5 II confocal microscope (Leica) equipped with 63× 1.4 numerical aperture oil objective in line‐scan mode at a rate of 5 ms per line, synchronized with the electrophysiological setup. Rhod‐2 was excited using the 543 nm line of the HeNe laser and fluorescence emission was collected at the wavelengths 560–660 nm. Dynamical Rhod‐2 fluorescence signal during Ca^2+^ transient was converted to [Ca^2+^]_i_ (Cheng *et al*. 1993), using the equation; [Ca^2+^]_i_ = *K*_d_ × (*F* – *F*_min_)/(*F*_max_ – *F*), where *K*_d_ Rhod‐2 = 1.58 μmol L^–1^ (Escobar *et al*. 1997; Trafford *et al*. 1995) and *F*_min_ = *F*_max_/15. *F*_max_ was determined by breaking the patch pipette and measuring the Rhod‐2 fluorescence as the dye was exposed to the 1 mmol L^–1^ Ca^2+^ bath solution. When there was a change in baseline fluorescence during the experiment, *F*/*F*_0_ was used in place of *F* in the calculation of [Ca^2+^]_i._ Submembrane [Ca^2+^] ([Ca^2+^]_sm_) was calculated based on (Weber *et al*. 2002). Once [Ca^2+^]_i_ was determined, the data were smoothed by the Savitzky–Golay method with 5 points of window. Then the data were differentiated and multiplied by a diffusion constant γ of 110 ms. These data were added to the [Ca^2+^]_i_ data to obtain the rise and peak of [Ca^2+^]_sm_. [Ca^2+^]_i_ and [Ca^2+^]_i_ + γ × *d*[Ca^2+^]_i_/dt were plotted and the decay fit with a single exponential such that it followed the decay of [Ca^2+^]_i_ (Fig. 13).

### *Ex vivo* optical mapping

Beating hearts were harvested from anaesthetized Sham and TAB rats via thoracotomy and were retrogradely perfused through the aorta in a Langendorff perfusion system (Radnoti Glass Technology, Monrovia, CA, USA) with 130 mmol L^–1^ NaCl, 24 mmol L^–1^ NaHCO_3_, 1.0 mmol L^–1^ MgCl_2_, 5.0 mmol L^–1^ KCl, 1.2 mmol L^–1^ NaH_2_PO_4_, 5 mmol L^–1^ dextrose and 1 mmol L^–1^ CaCl_2_ (pH 7.4), gassed with 95% O_2_ and 5% CO_2_. Constant flow perfusion was set to 10 mL min^–1^ with a peristaltic pump. Hearts were placed in a water‐heated chamber to maintain temperature at 37 ± 0.2°C and then 5 μmol L^–1^ blebbistatin was added to perfusate to reduce movement artefact. Hearts were stained with voltage sensitive indicator di‐4‐ANNEPS, using 20 μL of stock solution (1 mg mL^–1^ of DMSO) delivered through a bubble trap, above the aortic cannula. The ECGs were continuously monitored with a Powerlab system (AD Instruments, Sydney, NSW, Australia; RRID:SCR_001620). The optical apparatus has been described previously (Kim *et al*. 2015). Fluorescence images of APs were recorded from the anterior surface of the heart using a CMOS camera (100 × 100 pixels, 2000 frames s^–1^, 1.5 × 1.5 cm^2^ field of view; Ultima‐L; SciMedia, Costa Mesa, CA, USA). Hearts were stimulated with 150 ms cycle length and perfused with 50 nmol L^–1^ ISO and/or 10 nmol L^–1^ APA. Action potential durations (APDs) and conduction velocities were measured using *dF*/*dt* for activation and 75% of AP amplitude for repolarization by means of digital image analysis routines.

### Statistical analysis

Statistical analysis of electrophysiological, biochemical and Ca^2+^ imaging data was performed using Origin 8.0 (OriginLab Corp.; RRID:SCR_014212). Data are presented as the mean ± SD. Statistical significance between groups were performed using Student's *t* test (paired and unpaired) and one‐way ANOVA with a Bonferroni *post hoc* test where appropriate. *P* < 0.05 was considered statistically significant.

## Results

### Serine/threonine phosphorylation of SK2 channels is enhanced in hypertrophic rat ventricular myocytes

**Figure 1:**
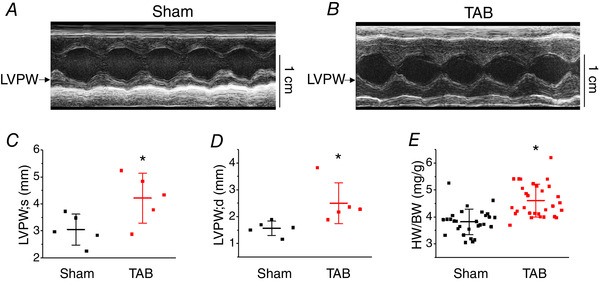
**Echocardiographic properties of Sham and TAB rat hearts** Representative echocardiographic M‐mode images in age‐matched Sham (*A*) and TAB rats (*B*) at the level of mitral valve. Left ventricular posterior wall (LVPW) dimensions of TAB rats are significantly increased in comparison to Sham, both in systole (*C*) and diastole (*D*). Mean ± SD of data indicated by line. *N* = 5 per group, ^*^ *P* = 0.04 (LVPW; s) and ^*^ *P* = 0.05 (LVPW; d), Student's *t* test. (*E*) Heart weight to body weight ratio of rats used in the present study. Mean ± SD of data indicated by line, Sham, *N* = 29; TAB, *N* = 30. ^*^ *P* < 0.001, Student's *t* test.

To investigate the regulatory mechanisms that control SK function, we used rats with hypertrophy induced by TAB. In this well‐established model, ligation of the ascending aorta induces pressure overload and the development of cardiac hypertrophy (del Monte *et al*. 2002; Wei *et al*. 2010). As determined by echocardiography, Fig. 1*A–D* shows that the left ventricular posterior wall thickness is significantly increased in the hearts of TAB rats 4 weeks after surgery, both in systole and diastole. The total heart weight/body weight ratio was 3.81 ± 0.09 (*n* = 29) and 4.61 ± 0.11^*^ (*n* = 30) mg g^–1^ for Sham and TAB respectively (^*^ *P* < 0.05, Student's *t* test, indicating significant heart enlargement in TAB animals. We have also previously demonstrated that, when challenged with β‐adrenergic agonist ISO (50 nmol L^–1^), 100% of *ex vivo* TAB rat hearts develop ventricular tachycardia/ventricular fibrillation compared to 15% of Shams (Kim *et al*. 2017).

**Figure 2:**
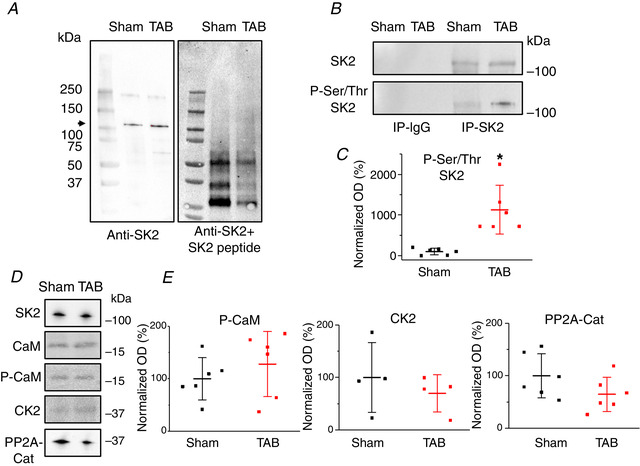
**Enhanced serine/threonine phosphorylation of SK2 in VMs from TAB rat hearts** *A*, SK2 channels immunoprecipitated from Sham and TAB VMs, with a band appearing at ∼100 kDa, as indicated by an arrow. This band is not present when anti‐SK2 and SK2 peptide are applied. *B*, SK2 channels immunoprecipitated from TAB VMs demonstrate increased serine/threonine phosphorylation. *C*, plot of optical density (OD) normalized to SK2 levels for phosphorylated‐serine/threonine SK2. Mean ± SD of data indicated by line. ^*^ *P* = 0.008 (P‐serine/threonine SK2), Student's *t* test. *D* and *E*, representative western blots and plots of OD normalized to SK2 levels for CK2, and PP2A‐C. Phosphorylated‐CaM (P‐CaM) was normalized to CaM in Sham and TAB rats, respectively. Mean ± SD of data indicated by line.

To gain insights into potential post‐translational changes in hypertrophy, we performed western blot analysis using immunoprecipitated SK2 complexes from freshly isolated Sham and TAB VMs (Fig. 2). Consistent with our previous report where anti‐SK2 antibodies were validated using shRNAs (Kim *et al*. 2017), SK2 protein bands were detected at ∼100 kDa (Fig. 2*A*), which is higher than the predicted molecular weight (62.2 KDa). Furthermore, protein bands at ∼100 kDa were not visible after application of anti‐SK2 peptide.

Probing with anti‐pan‐phospho‐serine/threonine antibody revealed significantly increased phosphorylation of SK2 channels in TABs *vs*. Shams (normalized optical density of Sham 100% ± 79.20% *vs*. TAB 1128% ± 602.18%, *n* = 6 per group) (Fig. 2*B* and *C*).

It was suggested that Ca^2+^ sensitivity of SK channels increases with dephosphorylation of associated CaM at the threonine‐79 site as a result of decreased levels of SK‐bound kinase CK2, or increased levels of phosphatase PP2A (Bildl *et al*. 2004; Yang *et al*. 2015; Zhang *et al*. 2014). Using anti‐phospho‐T79 CaM antibody, we did not find significant differences in phosphorylation of SK2‐bound CaM from TAB VMs compared to that from Sham VMs (Fig. 2*D* and *E*). Furthermore, we did not find significant changes in the abundance of SK‐tethered kinase CK2, or opposing PP2A‐C. These results imply CaM dephosphorylation probably does not serve as a mechanism for enhanced SK activity in our model of cardiac hypertrophy.

**Figure 3:**
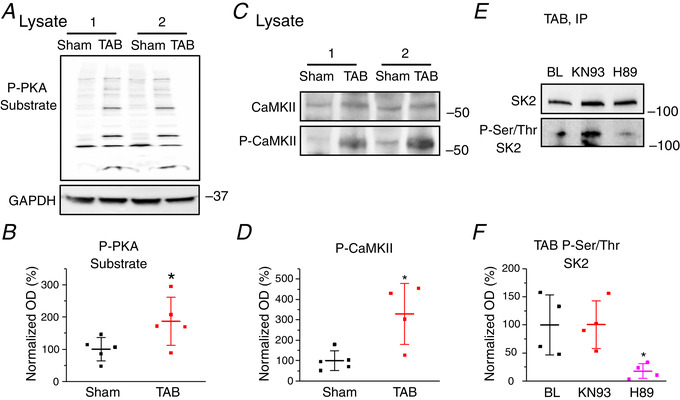
**Serine/threonine phosphorylation of SK2 in TAB rat hearts is PKA‐dependent** *A*, representative western blot demonstrating enhanced PKA‐dependent phosphorylation in TAB CMs. *B*, plot of optical density (OD) for phosphorylated‐PKA substrate normalized to GAPDH in Sham and TAB rat hearts, respectively. ^*^ *P* = 0.048, Student's *t *test. *C*, representative western blots demonstrating enhanced phosphorylation of CaMKII in VMs form TAB rats. *D*, plot of OD for phosphorylated‐CaMKII normalized to CaMKII in Sham and TAB rats. ^*^ *P* = 0.01, Student's *t *test. *E*, serine/threonine phosphorylation of immunoprecipitated SK2 in TAB hearts is reversible by inhibition of PKA (H89, 1 μmol L^–1^) but not inhibition of CaMKII (KN93, 500 nmol L^–1^). *F*, plot of OD for phosphorylated‐serine/threonine SK2 in TAB rats normalized to SK2 under normal conditions or treated with KN93 to block CaMKII activity or H89 to block PKA activity. ^*^ *P* = 0.03, one‐way ANOVA. BL, baseline. For all plots, mean ± SD of data indicated by line.

Next, we assessed potential changes in activities of two major serine/threonine kinases CaMKII and PKA in TABs *vs*. Shams using whole‐cell lysates from freshly isolated VMs. Experiments with anti‐phospho‐PKA substrate antibody (Fig. 3*A* and *B*) supported enhanced activity of the kinase in TABs *vs*. Shams. Figure 3*C* and *D* shows enhanced phosphorylation of CaMKII at threonine‐286, an indicator of CaMKII activation in hypertrophy (Tenma *et al*. 2018). To test which of these two kinases is responsible for SK hyperphosphorylation in hypertrophy, we incubated myocytes from TABs with pharmacological inhibitors of CaMKII, KN93 (500 nmol L^–1^) and PKA, H89 (1 μmol L^–1^) for 30 min. Subsequent probing of immunoprecipitated SK2 channels with anti‐serine/threonine antibodies (Fig. 3*E* and *F*) revealed a significant H89‐induced reduction of phosphorylation, whereas KN93 was ineffective. This suggests SK2 phosphorylation at serine/threonine sites is PKA‐ and not CaMKII‐dependent, which prompted us to next focus on the functional effects of PKA‐mediated phosphorylation on the channels.

### SK current in hypertrophic rat ventricular myocytes with preserved Ca^2+^ cycling

**Figure 4:**
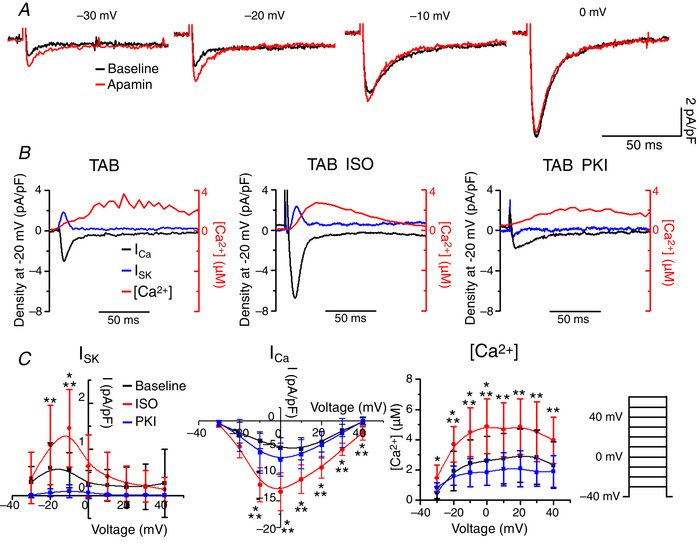
**PKA inhibition alleviates *I*_SK_ in TAB rat cardiomyocytes** *A*, representative traces of integral current before and after APA (100 nmol L^–1^) recorded at depolarizing steps with 10 mV intervals from HP ‐40 mV. *B*, representative superimposed traces of *I*_SK_ (blue), *I*_Ca_ (black) and [Ca^2+^]_i_ transient (red) recorded in TAB cells depolarized to −20 mV. *I*_SK_ is enhanced by β‐adrenergic stimulation with ISO (100 nmol L^–1^ for 3 min, centre) and decreased by PKA inhibitor PKI (1 μmol L^–1^) in the pipette solution. *C*, pooled mean ± SD *I*–*V* and peak [Ca^2+^]/*V* relationships for (*B*), with TAB cells at baseline (black), treated with ISO (red) and PKI (blue). *n* = 6–9, *N* = 5–8. *I*_SK_: ^*^ *P* = 0.01 (−10 mV) *vs*. TAB baseline. ^**^ *P* = 0.02 (−20 mV); *P* = 7 × 10^−4^ (−10 mV) *vs*. TAB PKI. *I*_Ca_: ^*^ *P* = 5.9 × 10^−5^ (−10 mV); *P* = 6.1 × 10^−5^ (0 mV); *P* = 5.7 × 10^−4^ (10 mV); *P* = 6.3 × 10^−4^ (20 mV), *P* = 0.002 (30 mV); *P* = 0.004 (40 mV) *vs*. TAB baseline. ^**^ *P* = 0.003 (−10 mV); *P* = 0.003 (0 mV); *P* = 0.007 (10 mV); *P* = 0.004 (20 mV); *P* = 0.01 (30 mV); *P* = 0.004 (40 mV) *vs*. TAB PKI. [Ca^2+^]: ^*^ *P* = 0.006 (−30 mV); *P* = 0.009 (−20 mV); *P* = 0.02 (−10 mV); *P* = 0.01 (0 mV) *vs*. TAB baseline. ^**^ *P* = 0.004 (−20 mV); *P* = 0.004 (−10 mV); *P* = 0.002 (0 mV); *P* = 0.01 (10 mV); *P* = 0.01 (20 mV); *P* = 0.02 (30 mV); *P* = 0.03 (40 mV) *vs*. TAB PKI, one‐way ANOVA with a Bonferroni *post hoc* test.

To isolate the SK current in TAB VMs, patch clamped VMs were held at −40 mV to inactivate Na^+^ and most of voltage‐dependent K^+^ currents. Intracellular [Ca^2+^] was measured simultaneously using line‐scan laser confocal imaging with 100 μmol L^–1^ Ca^2+^ indicator Rhod‐2 in the pipette solution. Under these conditions, integral currents upon depolarizing wsteps at 10 mV increments are predominantly carried by LTCCs and SK channels that lack voltage‐dependent inactivation. Figure 4*A* demonstrates representative recordings of integral current before (black traces) and after application of specific peptide SK inhibitor APA (100 nmol L^–1^ for 3 min, red) (Stocker, 2004). The SK current (*I*_SK_) was obtained by subtraction of traces before and after channel inhibition by APA. Figure 4*B* shows superimposed traces of *I*_SK_ (blue), *I*_Ca_ (black, i.e. residual integral current after APA) and *I*_Ca_‐induced cytosolic Ca^2+^ transient derived from confocal line‐scan imaging recording of Rhod‐2 signal (in red) evoked by a depolarizing step to −20 mV. As seen in Fig. 4*B*, the APA‐sensitive current rapidly activates and inactivates when [Ca^2+^]_i_ still continues to rise, confirming biphasic regulation of SK channels by Ca^2+^ in native myocytes. Furthermore, the current–voltage (*I*–*V*) relationship data presented in Fig. 4*C* demonstrates that *I*_SK_ peaks at −20 mV (left, black) and declines at higher voltages, whereas amplitudes of both *I*_Ca_ and Ca^2+^ transients continue to rise (Fig. 4*C*, centre and right, respectively). These data suggest that, in native VMs, SK channels activated by submicromolar [Ca^2+^] during Ca^2+^ transient can be effectively inhibited in a voltage‐dependent manner when [Ca^2+^]_i_ reaches supramicromolar concentrations.

Next, we aimed to test whether changes in PKA activity can modulate *I*_SK_. Incubation of TAB VMs with the β‐adrenergic agonist ISO (100 nmol L^–1^ for 3 min) increased *I*_SK_ amplitude and shifted the *I*–*V* curve to the right in parallel to an increase in *I*_Ca_ and Ca^2+^ transients (Fig. 4*B*, centre, and Fig. 4*C*, red lines). By contrast, inhibition of PKA by the specific peptide inhibitor PKI (1 μmol L^–1^) introduced into patch‐pipette solution diminished *I*_SK_ in TAB VMs without significantly altering *I*_Ca_ and Ca^2+^ transient amplitudes (Fig. 4*B*, right, and Fig. 4*C*, blue lines). These data strongly suggest that PKA phosphorylation of SK channels in VMs from TABs positively modulates their activity, possibly lessening the voltage‐dependent inhibition of the channels that occurs at higher concentrations of intracellular Ca^2+^.

**Figure 5:**
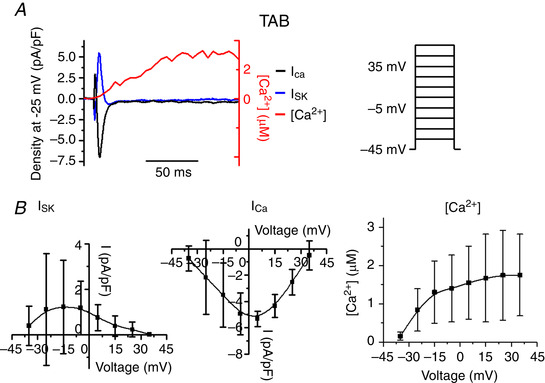
**SK current in TAB rat VMs obtained by application of UCL‐1684** *A*, representative superimposed traces of *I*_SK_ (blue), *I*_Ca_ (black) and [Ca^2+^]_i_ transient (red) recorded in TAB VMs depolarized to −25 mV from −45 holding potential. *I*_SK_ was obtained after subtraction of the current after application of 1 μmol L^–1^ UCL‐1684. Stimulus voltage protocol (right). *B*, pooled mean ± SD *I*–*V* and peak [Ca^2+^]/*V* relationships for (*A*). *n* = 5, *N* = 5.

Although Yu *et al*. 2014 previously demonstrated that apamin at concentrations of up to 500 nmol L^–1^ does not affect Na^+^, Ca^2+^ and other major K^+^ currents, concern remains that 100 nmol L^–1^ apamin can exert non‐specific effects. To address this issue, we used the non‐peptidic inhibitor of SK channels UCL‐1684 (Hosseini *et al*. 2001; Rosa *et al*. 1998) to isolate *I*_SK_ in TAB myocytes. Using 1 μmol L^–1^ UCL‐1684, we obtained results similar to those with 100 nmol L^–1^ APA (Fig. 5).

**Figure 6:**
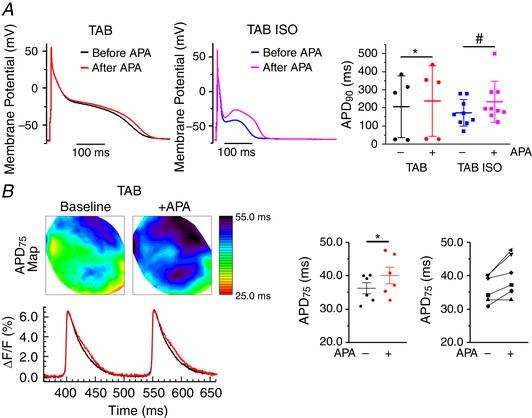
**Apamin prolongs action potentials in TAB rat VMs and TAB rat whole heart** *A*, representative current clamp traces in TAB rat VMs under baseline (left) or ISO (100 nmol L^–1^ for 3 min) (right) before and after treatment with apamin (100 nmol L^–1^ for 3 min). Pooled mean ± SD APD_90_. *n* = 5–9, *N* = 4–7, ^*^ *P* = 0.04, #*P* = 0.03, Student's *t* test. *B*, representative APD maps before and after APA (10 nmol L^–1^) recorded under basal conditions and in the presence of β‐adrenergic agonist ISO (50 nmol L^–1^) and the corresponding APD profile. Right, Pooled data for APD_75_. ^*^ *P* < 0.05, paired Student's *t* test, *N* = 6.

Current clamp experiments demonstrated that APA prolongs APD in myocytes from TABs paced at stimulation frequency 0.5 Hz under basal conditions and in the presence of ISO (100 nmol L^–1^) (Fig. 6*A*). These data confirm that, in disease, SK channels contribute to repolarization (Chua *et al*. 2011; Skibsbye *et al*. 2014) Similarly, in optically mapped *ex vivo* TAB hearts stained with voltage sensitive dye di‐4‐ANNEPS, 10 nmol L^–1^ APA prolonged APD under basal conditions (Fig. 6*B*). Unfortunately, we were unable to assess potential APA‐dependent changes in APD under β‐adrenergic stimulation because, in all of the hearts studied, perfusion with ISO evoked ventricular tachycardia/ventricular fibrillation, which is consistent with our previous report (Kim *et al*. 2017).

### β‐adrenergic stimulation evokes SK current in Sham rat VMs

**Figure 7:**
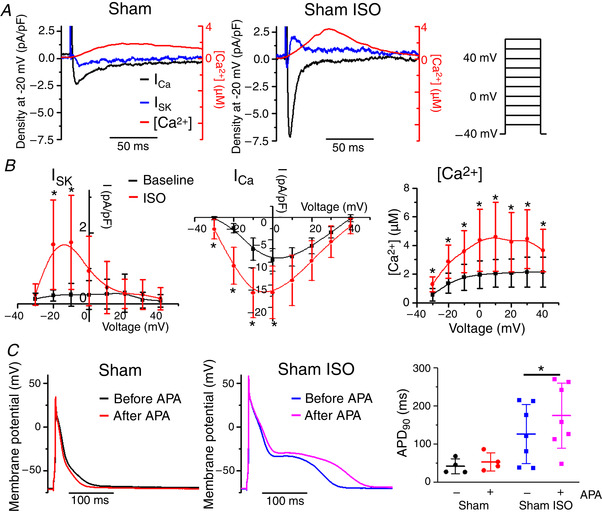
**β‐adrenergic stimulation evokes *I*_SK_ in Sham rat VMs** *A*, representative superimposed traces of *I*_SK_ (blue), *I*_Ca_ (black) and [Ca^2+^]_i_ transient recorded in TAB VMs depolarized to –20 mV. *I*_SK_ is enhanced by β‐adrenergic stimulation with ISO (100 nmol L^–1^). *B*, pooled *I*–*V* and peak [Ca^2+^]/*V* relationships for (*A*), with Sham VMs at baseline (black) and treated with ISO (red). Mean ± SD, *n* = 5–11, *N* = 5–8. *I*_SK_: ^*^ *P* = 0.03 (−20 mV), *P* = 0.03 (−10 mV) *vs*. baseline; *I*_Ca_: ^*^ *P* = 0.03 (−30 mV), *P* = 7.7 × 10^−4^ (−20 mV), *P* = 0.003 (−10 mV), *P* = 0.02 (0 mV) *vs*. baseline. [Ca^2+^]: ^*^ *P* = 0.006 (−30 mV), *P* = 0.004 (−20 mV), *P* = 0.007 (−10 mV), *P* = 0.01 (0 mV), *P* = 0.01 (10 mV), *P* = 0.01 (20 mV), *P* = 0.01 (30 mV), *P* = 0.03 (40 mV) *vs*. baseline, one‐way ANOVA with a Bonferroni *post hoc* test. *C*, representative APD traces recorded before and after application of apamin (APA, 100 nmol L^–1^ for 3–6 min) under baseline conditions and in the presence of ISO (100 nmol L^–1^). Right: pooled data for APD_90_. Mean ± SD of data indicated by line, *n* = 4–7, *N* = 4–6. ^*^ *P* = 0.002, paired Student's *t* test.

It has remained unclear why APD and currents in healthy VMs from humans and various animal models are insensitive to APA, despite immunodetection of endogenous SK channels (Bonilla *et al*. 2014; Chua *et al*. 2011; Gui *et al*. 2012; Hsieh *et al*. 2013; Nagy *et al*. 2011). We reasoned that, if PKA‐dependent phosphorylation does indeed play a major role in the modulation of SK channel function, we should be able to evoke SK current to affect APD by β‐adrenergic stimulation in freshly isolated VMs from Sham hearts. As shown in Fig. 7*A* and *B*, application of ISO (100 nmol L^–1^ for 3 min) increased *I*_Ca_ and Ca^2+^ transients and evoked *I*_SK_ in VMs from Sham rat hearts, which was otherwise undetectable under basal conditions. The *I*–*V* curve in Fig. 7*B* (left) reveals an ISO‐evoked APA‐sensitive current with characteristics resembling *I*_SK_ from ISO‐stimulated TAB VMs (Fig. 4*C*, left, red), with a peak at −10 mV. Figure 7*C* demonstrates representative recordings of APs in current clamped VMs from Shams before and after APA application (100 nmol L^–1^ for 3 min). In line with previous studies in healthy VMs under basal conditions (Chua *et al*. 2011; Gui *et al*. 2012), application of APA produced no effects. On the other hand, in the presence of ISO (100 nmol L^–1^), APA significantly prolonged APD.

**Figure 8:**
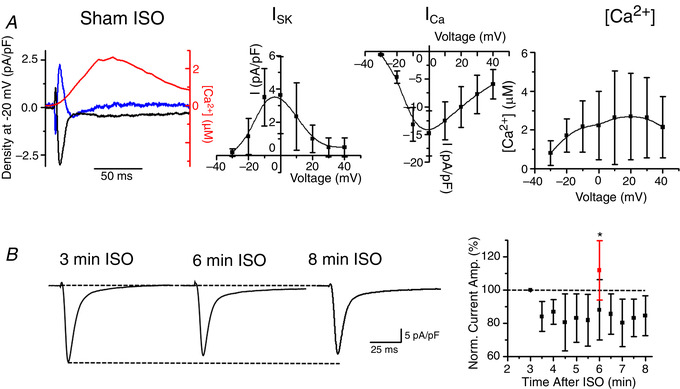
**SK current in Sham rat VMs with application of ISO** *A*, representative superimposed traces of *I*_SK_ (blue), *I*_Ca_ (black) and [Ca^2+^]_i_ transient (red) recorded in Sham VMs depolarized to −20 mV (left). *I*_SK_ was obtained by application of 1 nmol L^–1^ APA. Pooled *I*–*V* and peak [Ca^2+^]/*V* relationships. *B*, representative inward current traces recorded in Sham VMs at 3, 6 and 8 min following application of 100 nmol L^–1^ ISO at −10 mV (HP −40 mV). Right: plot of normalized inward current amplitude *vs*. time after application of ISO. Mean ± SD, *n* = 5, *N* = 2. The normalized inward current after 6 min of ISO and 3 min of 100 nmol L^–1^ apamin is plotted in red. Mean ± SD, *n* = 8, *N* = 7, ^*^ *P* = 0.04 *vs*. ISO only, Student's *t* test.

The currents derived using 100 nmol L^–1^ APA or 1 μmol L^–1^ UCL‐1684 are relatively small: ∼1–4 pA pF^–1^ (Figs 4 and 5, respectively). Figure 8 depicts additional control experiments corroborating our findings. Figure 8 *A* demonstrates representative traces and pooled data for *I*_Ca_, Ca^2+^ transients and *I*_SK_ derived using 1 nmol L^–1^ APA in Sham myocytes challenged with ISO (100 nmol L^–1^). A lower concentration of APA produces similar results to those when 100 nmol L^–1^ APA is used (Fig. 7), which is consistent with the results of a study by Yu *et al*. 2014 showing that the latter APA concentration does not affect other major ionic currents in the cardiomyocytes.

Figure 8*B* demonstrates time‐dependent changes in inward current of ISO‐treated myocytes depolarized to −10 mV from a holding potential of −40 mV. The time point used to apply SK channel antagonists APA or UCL‐1684 was 3 min after ISO and *I*_SK_ was derived from recordings obtained 3–5 min after application of these drugs. Therefore, inward current amplitude recorded 3 min after application of ISO was considered as 100%. Between 6 and 8 min after application of the ISO time window, there was a ∼20% decrease in inward current, probably as a result of *I*_Ca_ rundown. Importantly, pooled data show that, in the presence of 100 nmol L^–1^ APA, integral inward current amplitude increases, which is consistent with the block of outward component (i.e. *I*_SK_).

**Figure 9:**
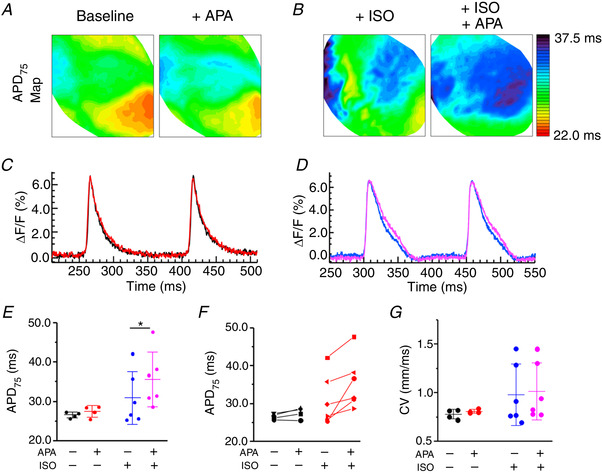
***I*_SK_ inhibition prolongs APD in *ex vivo* Sham rat hearts under β‐adrenergic‐stimulation** *A* and *B*, representative APD maps before and after APA (10 nmol L^–1^) recorded under basal conditions and in the presence of β‐adrenergic agonist ISO (50 nmol L^–1^) and the corresponding APD profiles *C*–*F*, pooled data for APD75. *G*, pooled data for conduction velocity (CV). ^*^ *P* < 0.05, paired Student's *t* test, *N* = 4–6.

To extend our cell studies to the tissue level, as shown in Fig. 6, we performed optical mapping experiments in *ex vivo* hearts from Sham rats stained with the voltage sensitive indicator di‐4‐ANNEPS. Representative APD maps of Langendorff‐perfused Sham hearts under basal conditions and after incubation with 50 nmol L^–1^ ISO are shown in Fig. 9*A* and *B*. APD maps and corresponding AP traces (Fig. 9*C* and *D*) demonstrate that 30 min of perfusion with APA (10 nmol L^–1^) has no effects under baseline but evokes significant prolongation of repolarization in the presence of ISO (Fig. 9*E* and *F*). Of note, APA did not affect conduction velocity (Fig. 9*G*).

### Biphasic regulation of native SK channels by [Ca^2+^]

**Figure 10:**
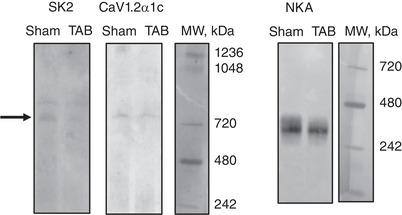
**BN‐PAGE reveals a physical interaction of SK2 and the α1c subunit of LTCC in Sham and TAB rat VMs** Samples for BN‐PAGE were prepared from the membrane fraction of freshly isolated Sham and TAB rat VMs. SK2 and the α1c subunit of LTCC can be detected at the same band, indicative of a physical interaction between these two proteins. NKA was used as a loading control. *N* = 3 per group.

**Figure 11:**
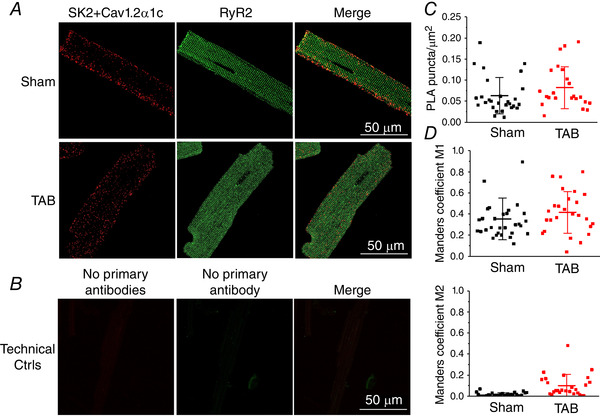
**PLA reveals low level colocalization of SK2‐Cav1.2α1c complexes with RyR2 in Sham and TAB rat VMs** *A*, PLA of SK2 and Cav1.2α1c, and colocalization with RyR2. Left: each red fluorescent dot represents a site of interaction of proteins that are within 40 nm proximity. Centre: cells were probed with anti‐RyR2 antibody. Right: merged image showing colocalization of SK2 protein pairs with RyR2. *B*, PLA and RyR2 secondary antibodies only, as a technical control. *C*, quantification of the number of PLA puncta per μm^2^ in Sham and TAB VMs. No significant differences were observed between groups, Student's *t *test, Sham *n* = 29, TAB *n* = 28, *N* = 4 per group. *D*, Manders coefficients M1 and M2 reveals a low level of colocalization of SK2‐Cav1.2α1c with RyR2. No significant differences were observed between groups Student's *t *test: Sham, *n* = 31; TAB, *n* = 26; *N* = 4 per group.

**Figure 12:**
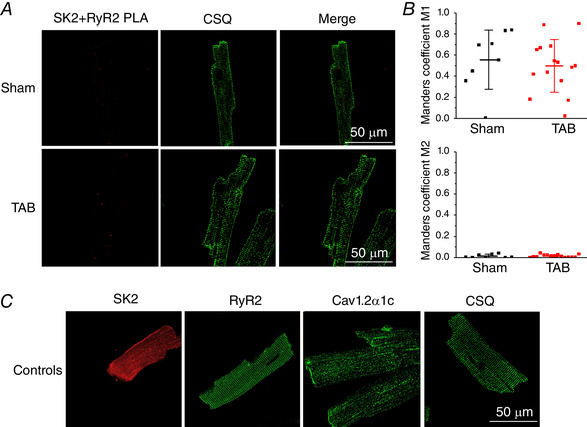
**PLA reveals low level colocalization of SK2‐RyR2 complexes with CSQ in Sham and TAB rat VMs** *A*, PLA of SK2 and RyR2 and colocalization with CSQ. Left: each red fluorescent dot represents a site of interaction of proteins that are within 40 nm proximity. Centre: cells were probed with anti‐CSQ antibody. Right: merged image showing colocalization of SK2 protein pairs with RyR2. *B*, Manders coefficients M1 and M2 reveals a low level of colocalization of SK2‐RyR2 with CSQ. No significant differences were observed between groups. Student's *t* test: Sham, *n* = 8; TAB, *n* = 15; *N* = 3 per group. *C*, SK2, RyR2, Cav1.2α1c and CSQ primary antibodies without PLA protein pairing.

Previous studies using heterologous expression systems demonstrated that, in addition to being activated by Ca^2+^ in the submicrocmolar range, SK channels are sensitive to voltage‐dependent inhibition by Ca^2+^ (IC_50_ ∼20 μmol L^–1^) (Soh & Park, 2002). During Ca^2+^ release from the SR, [Ca^2+^] rapidly increases from ∼100 nmol L^–1^ up to 100 μmol L^–1^ in specific subcellular compartments in close proximity to RyR clusters (i.e. dyads) (Antoons *et al*. 2011; Cannell *et al*. 2013). Therefore, localization of SK channels relative to the sources of Ca^2+^ is expected to determine the *I*_SK_ kinetics of activation and inactivation. Assessment of the exact localization of SKs on surface membrane using imaging techniques is confounded by the quality of existing antibodies and the presence of these channels in other cellular compartments including mitochondria (Kim *et al*. 2017). Lu *et al*. (2007) demonstrated that SK channels in mouse myocytes exist in complexes with LTCCs. Using the BN‐PAGE technique, where mild processing of samples allows many protein complexes to remain intact, we confirmed that, in Sham and TAB rat VMs, SK2 and the α1c subunit of LTCC can be detected in the same band, indicative of complex formation (Fig. 10, SK2‐LTTC α1c complex indicated by black arrow). Samples for BN‐PAGE were prepared from the membrane fraction of freshly isolated VMs and Na^+^/K^+^‐ATPase was used as the loading control. These results supported the specific tagging of SK2 channels present in plasmalemma in complex with LTCCs using PLA (Fig. 11). To stain fixed VMs from Shams and TAB rats, we used primary antibodies against SK2, LTCC α1c and RyR2. Secondary antibodies for RyR2 were labelled with fluorescein (green, Alexa‐488). Secondary antibodies for SK2 and LTCC were conjugated with complementary DNA strands that form a red fluorescent tag after processing if in close proximity (i.e. less than 40 nm). Technical control experiments included samples processed for the entire PLA and colocalization assay but without incubation with primary antibodies (Fig. 11*B*). The density of SK2‐LTCC complexes visualized using PLA was not statistically different in Shams *vs*. TABs (Fig. 11*C*). Importantly, analysis of confocal immunofluorescence images indicated that colocalization of SK2‐LTCC complexes with RyR2 clusters in TABs and Shams is low (Fig. 11*D*), suggesting that the majority of plasmalemmal SK channels are situated outside the dyad. To test this further, we tagged SK2 channels in complex with RyR2 using PLA, and assessed the colocalization of these protein pairs with calsequestrin to visualize junctional SR (CSQ) (Fig. 12). The overall density of SK2‐RyR2 pairs was significantly lower than density of SK2‐LTCC pairs (0.005 ± 0.005 SK2‐RyR2 *vs*. 0.063 ± 0.043 SK2‐LTCC PLA puncta μm^–2^ in Sham VMs, *P* < 0.005, Student's *t *test; and 0.023 ± 0.020 SK2‐RyR2 *vs*. 0.101 ± 0.067 SK2‐LTCC PLA puncta μm^–2^ in TAB VMs, *P* < 0.005, Student's *t *test). Manders coefficients of colocalization were also low, suggesting that the very few SK2‐RyR2 pairs visualized by the PLA technique do not reside in the dyad. Figure 12*C* shows representative control images of VMs stained by anti‐SK2, anti‐Cav1.2a1c, anti‐RyR2 and anti‐CSQ antibodies without PLA.

**Figure 13:**
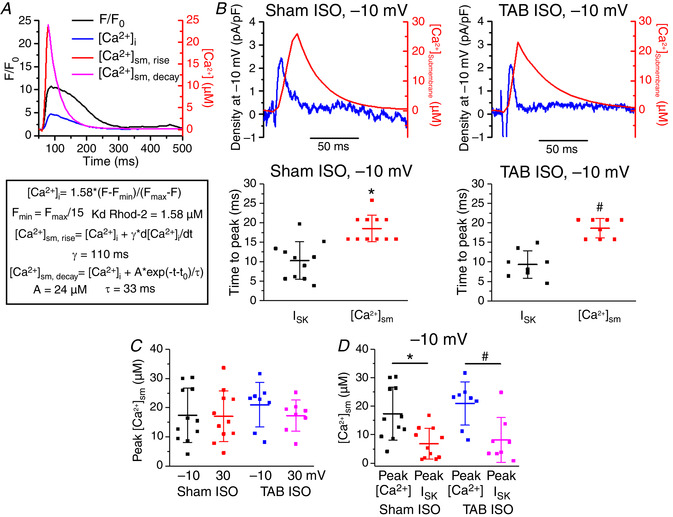
***I*_SK_ peaks, whereas submembrane [Ca^2+^] continues to rise, indicating an inhibition of *I*_SK_ by Ca^2+^** *A*, sample traces and equations for calculating submembrane Ca^2+^ concentration ([Ca^2+^]_sm_) based on calculations used previously (Weber *et al*. 2002). *B*, top: representative traces of *I*_SK_ (blue) and [Ca^2+^]_sm_ (red) at a –10 mV voltage step under ISO stimulation (100 nmol L^–1^). Bottom: plots of the time to peak for *I*_SK_ and [Ca^2+^]_sm_ in Sham and TAB myocytes. Line indicates mean ± SD. ^*^ *P* = 1.7 × 10^−4^, *n* = 11, *N* = 8, Student's *t *test. #*P* = 2.4 × 10^−5^, *n* = 8, *N* = 7, Student's *t *test. *C*, plot of peak [Ca^2+^]_sm_ at −10 and 30 mV voltage steps under ISO stimulation (100 nmol L^–1^ for 3 min). Line indicates mean ± SD. There is no significant difference in any of the peak [Ca^2+^]_sm_ values, *P* = 1, one‐way ANOVA, with a Bonferroni *post hoc* test. *D*, plot comparing the [Ca^2+^]_sm_ at the peak [Ca^2+^] to [Ca^2+^]_sm_ at peak *I*_SK_ for Sham and TAB myocytes under ISO stimulation. Line indicates mean ± SD. ^*^ *P* = 0.001, *n* = 11, *N* = 8, paired Student's *t* test. #*P* = 0.003, *n* = 8, *N* = 7, paired Student's *t* test.

An absence of SK channels in the immediate proximity of SR Ca^2+^ release channel clusters allowed us to employ mathematical apparatus developed by Trafford *et al*. (1995) and Weber *et al*. (2002) for estimation of [Ca^2+^] in the subsarcolemmal compartment from the fluorescence signal of Ca^2+^ indicator Rhod‐2 during the Ca^2+^ transient (Fig. 13*A*). Fluorescence was converted into bulk [Ca^2+^]_i_. To assess the rapid rising phase, we differentiated the [Ca^2+^]_i_ and multiplied by a diffusion constant γ of 110 ms (Weber *et al*., 2002). The result was then added to [Ca^2+^]i to determine the rise and peak of submembrane Ca^2+^ ([Ca^2+^]_sm_). To estimate the decay of [Ca^2+^]_sm_, the [Ca^2+^]_i_ + γ*d[Ca^2+^]_i_/dt trace was fit with a single exponential curve using the decay of [Ca^2+^]_i_ as a guide to fitting (Fig. 13*A*). Figure 13*B* depicts superimposed representative traces of *I*_SK_ and [Ca^2+^]_sm_ in ISO‐treated voltage clamped Sham and TAB VMs recorded during depolarization from holding potential of −40 mV to −10 mV. *I*_SK_ was obtained from current recording before and after application 100 nmol L^–1^ APA as described above. Importantly, *I*_SK_ at −10 mV reaches the peak significantly earlier than [Ca^2+^]_sm_, which confirms that Ca^2+^ not only activates native SK channels, but also inhibits SK channels at higher concentrations. The pooled data for *I*_SK_ and [Ca^2+^]_sm_ time to peak are shown in Fig. 13*B*. Also, at higher 30 mV voltage pulses, *I*_SK_ current is negligible despite little change in [Ca^2+^]_sm_ amplitude (Fig. 13*C*). Peak [Ca^2+^]_sm_ amplitude and [Ca^2+^]_sm_ at peak *I*_SK_ are presented in Fig. 13*C* and *D*. The data suggest that, in the presence of ISO, *I*_SK_ amplitude reaches a maximum when [Ca^2+^]_sm_ on average rises to ∼10 μmol L^–1^ in both Sham and TAB VMs. The continuing rise of [Ca^2+^]_sm_ above this level effectively elicits *I*_SK_ inactivation. The absence of *I*_SK_ at higher voltages at similar amplitudes of [Ca^2+^]_sm_ suggests strong voltage‐dependence of SK channel block by Ca^2+^. Notably, in the absence of ISO, [Ca^2+^]_sm_ in Sham VMs at −10 mV was 10.4 ± 6.5 μmol L^–1^ (mean ± SD, *n* = 10, *N* = 6) and TAB VMs (10 mV) was 7.6 ± 5.1 μmol L^–1^ (mean ± SD, *n* = 8, *N* = 6) which is within the previously reported activation range for SK channels (Li N *et al*. 2009; Li W *et al*. 2009; Schumacher *et al*. 2001; Schumacher *et al*. 2004; Soh & Park, 2002; Xia *et al*. 1998). These data suggest that β‐adrenergic stimulation evokes *I*_SK_ in freshly isolated VMs by lessening Ca^2+^ dependent block.

### β‐adrenergic stimulation reduces Ca^2+^/voltage‐dependent inhibition of SK2 channels without affecting activation by [Ca^2+^]

**Figure 14:**
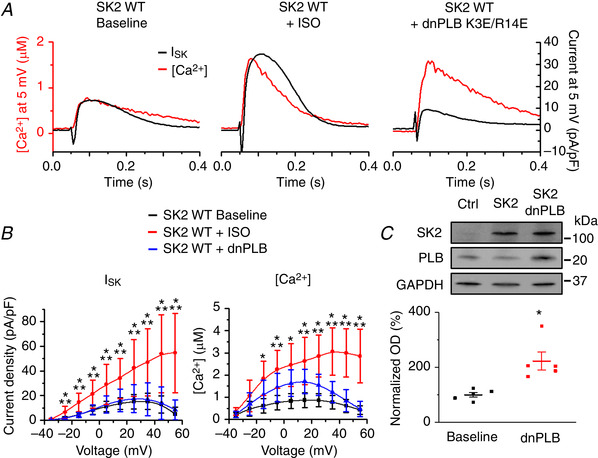
**Effects of β‐adrenergic stimulation on *I*_SK_ in cultured rat VMs overexpressing rSK2** *A*, Representative traces of [Ca^2+^]_i_ transients and *I*_SK_ before and after application of ISO (100 nmol L^–1^) in control rSK2‐overexpressing VMs and VMs co‐expressing dnPLB. VMs were cultured with adenoviral expression vectors for 48 h. *B*, pooled *I*–*V* and [Ca^2+^]/*V* relationships for (*A*). Mean ± SD, *n* = 8–10, *N* = 4–5. *I*_SK_: ^*^ *P* = 0.002 (−35 mV), *P* = 0.003 (−25 mV), *P* = 2.6 × 10^−4^ (−15 mV), *P* = 0.002 (−5 mV), *P* = 0.003 (5 mV), *P* = 0.002 (15 mV), *P* = 0.002 (25 mV), *P* = 0.003 (35 mV), *P* = 0.001 (45 mV), *P* = 6.3 × 10^−5^ (55 mV) *vs*. baseline. ^**^ *P* = 0.002 (−35 mv), *P* = 0.002 (−25 mV), *P* = 1.6 × 10^−4^ (−15 mV), *P* = 0.002 (−5 mV), *P* = 0.006 (5 mV), *P* = 0.005 (15 mV), *P* = 0.004 (25 mV), *P* = 0.004 (35 mV), *P* = 8.7 × 10^−4^ (45 mV), *P* = 8.5 × 10^−5^ (55 mV) *vs*. dnPLB. [Ca^2+^]: ^*^ *P* = 0.001 (−15 mV), *P* = 3.2 × 10^−4^ (−5 mV), *P* = 2.3 × 10^−4^ (5 mV), *P* = 1.7 × 10^−4^ (15 mV), *P* = 4.3 × 10^−6^ (25 mV), *P* = 2.3 × 10^−6^ (35 mV), *P* = 1.6 × 10^−6^ (45 mV), *P* = 1.8 × 10^−6^ (55 mV) *vs*. baseline. ^**^ *P* = 0.04 (−5 mV), *P* = 0.04 (15 mV), *P* = 5.8 × 10^−4^ (25 mV), *P* = 2.7 × 10^−5^ (35 mV), *P* = 3.6 × 10^−6^ (45 mV), *P* = 1.1 × 10^−6^ (55 mV) *vs*. dnPLB, one‐way ANOVA with a Bonferroni *post hoc* test. *C*, representative western blots from VMs probed for SK2 and PLB. The control lane (Ctrl) represents VMs infected with virus carrying an empty vector, the SK2 lane represents VMs infected with a WT SK2 virus, and the SK2 dnPLB lane represents VMs infected with WT SK2 and dnPLB viruses. Mean ± SD optical density normalized to GAPDH for SK2 at baseline is 100 ± 58.9 and SK2 + dnPLB is 150.3 ± 22.6. Mean ± SD optical density for PLB at baseline is 99.99 ± 19.8 and for SK2 + dnPLB 222.6 ± 73.04, which is significantly different compared to baseline, *P* = 0.02, Student's *t* test, *N* = 5.

To gain mechanistic insights into the roles of PKA in modulation of SK function, we used the well‐established experimental system of cultured rat VMs. In line with our previous report (Terentyev *et al*. 2014), adenovirus‐mediated overexpression of rSK2 in cultured VMs produced a robust *I*_SK_ between 36 and 48 h after infection with a multiplicity of infection of 10. As seen in Fig. 14*A* and *B*, application of ISO enhanced *I*_SK_ in parallel with the increase in Ca^2+^ transient amplitudes in voltage clamped VMs (holding potential −45 mV). This effect was more pronounced at higher depolarizations resulting in ISO‐mediated loss of rectification, as seen in respective current‐voltage relationships (Fig. 14*B*, left, red *vs*. black). To distinguish between the possible effect of increasing [Ca^2+^]_i_ and the direct effect of PKA‐mediated phosphorylation of SK channels, rSK2 was coexpressed with dnPLB (K3E/R14E mutation) (Ziolo *et al*. 2005). Adenoviral‐mediated expression of each protein was confirmed by western blot analysis using VMs infected with adenovirus carrying an empty vector as a control (Fig. 14*C*). Coexpression of dnPLB relieves inhibition of SERCa2a resulting in higher Ca^2+^ transients during voltage pulses of the experiment, without increasing PKA activity. Importantly, despite the dnPLB‐mediated increase in intracellular Ca^2+^ transient amplitude, this was not accompanied by an increase in *I*_SK_. Increased Ca^2+^ transient amplitude alone did not increase *I*_SK_ at 5 mV compared to baseline (Fig. 14*A*, left *vs*. right) and did not lessen the rectification of *I*_SK_ as observed in the ISO‐treated group (Fig. 14*B*, blue *vs*. red line). These results are consistent with the hypothesis that PKA phosphorylation enhances *I*_SK_ predominantly via reduction of voltage‐dependent inhibition of the channels by [Ca^2+^]_i_.

**Figure 15:**
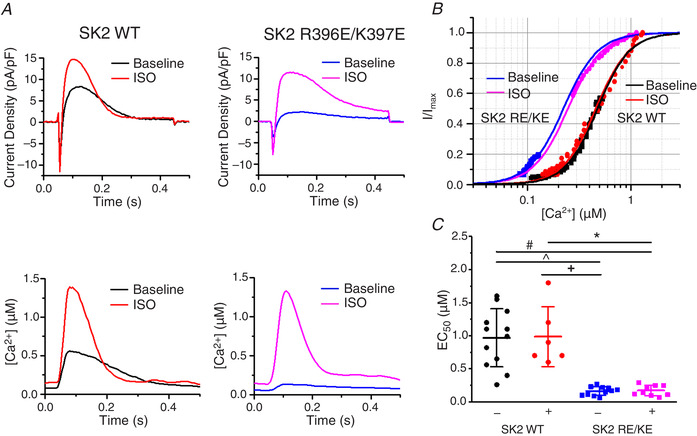
**β‐adrenergic stimulation of *I*_SK_ in cultured rat VMs does not shift Ca^2+^ sensitivity** *A*, representative traces of *I*_SK_ and [Ca^2+^]_i_ transients before and after application of ISO (100 nmol L^–1^) in WT rSK2‐overexpressing VMs and VMs expressing the rSK2 double mutant R396E/K397E. VMs were cultured with adenoviral expression vectors for 48 h. *B*, SK current dependence on [Ca^2+^] for one WT rSK2 expressing VM and one rSK2 double mutant expressing VM. Current was normalized to the peak current in the presence of the β‐adrenergic agonist ISO (100 nmol L^–1^, *I*_max_) and plotted from the peak through decay to minimum. Data was fitted with a Hill equation *I*/*I*_max_ = 1/[1 + (EC_50_/[Ca^2+^])^h^] where *h* is the Hill coefficient. *C*, plot of average EC_50_ values for WT rSK2 and the rSK2 double mutant recorded under basal conditions and in the presence of the β‐adrenergic agonist ISO (100 nmol L^–1^). Mean ± SD of data indicated by line, *n* = 6–12, *N* = 4‐5. ^*P* = 2.4 × 10^−6^, #*P* = 7.9 × 10^−6^, +*P* = 4.9 × 10^−5^, ^*^ *P* = 1.0 × 10^−4^, one‐way ANOVA with a Bonferroni *post hoc* test.

Previous studies ascribed *I*_SK_ enhancement in VMs from diseased hearts to the leftward shift of sensitivity of channels to activating [Ca^2+^]_i_ with a decrease in EC_50_ from ∼600 nmol L^–1^ to 200 nmol L^–1^ (Chang *et al*. 2013b; Gui *et al*. 2012). To assess the possible effects of PKA on SK sensitivity to activating [Ca^2+^], we used Ca^2+^ transients evoked by depolarization to 5 mV as a ramp of [Ca^2+^] during simultaneous recording of *I*_SK_. Figure 15*A* shows superimposed representative traces of *I*_SK_ recorded before and after application of ISO (100 nmol L^–1^ for 3 min, black and red, respectively) and corresponding Ca^2+^ transients (Fig. 15*A*, lower). The analysis that followed used the decaying phase of Ca^2+^ transient where [Ca^2+^]_i_ matches [Ca^2+^]_sm_ after the peak of *I*_SK_, which also allowed us to avoid contamination with *I*_Ca_. In Fig. 15*B* normalized SK currents from the same VM are plotted against [Ca^2+^]_i_ where the peak amplitude of *I*_SK_ under ISO was considered as *I*_Max_. Importantly, curve fitting using Hill's equation produced similar results for data obtained under baseline conditions and after challenge with ISO showing no change in EC_50_ to [Ca^2+^] (baseline EC_50_ = 460 nmol L^–1^, *h* = 2.6; ISO EC_50_ = 480 nmol L^–1^, *h* = 2.9). To validate our approach, we performed similar analysis in VMs overexpressing an rSK2 mutant with enhanced sensitivity to [Ca^2+^]_i_ (R396E/K397E, EC_50_ = 200 nmol L^–1^, Li & Aldrich, 2011). Representative current recordings (Fig. 15*A*, blue and magenta) demonstrated a visibly slower decay kinetics of the RE/KE mutant, which translated into a significant shift in the current/[Ca^2+^] relationship to the left (baseline EC_50_ = 230 nmol L^–1^, *h* = 2.5; ISO EC_50_ = 240 nmol L^–1^, *h* = 2.5) (Fig. 15*B*). Pooled data for EC_50_ are presented in Fig. 15*C*. These results further confirm that PKA phosphorylation does not change the affinity of SK channels to activating submicromolar [Ca^2+^] but, instead, reduces Ca^2+^/voltage‐dependent inhibition at higher levels of intracellular Ca^2+^.

### Serine‐465 confers PKA‐mediated regulation of rSK2 activity

**Figure 16:**
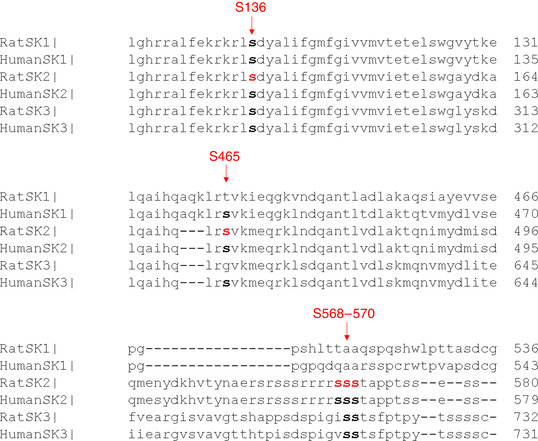
**Protein sequence alignment of rat and human SK isoforms showing putative PKA phosphorylation sites** Potential PKA phosphorylation sites of SK2, as suggested in Ren *et al*. (2006) are highlighted in red.

**Figure 17:**
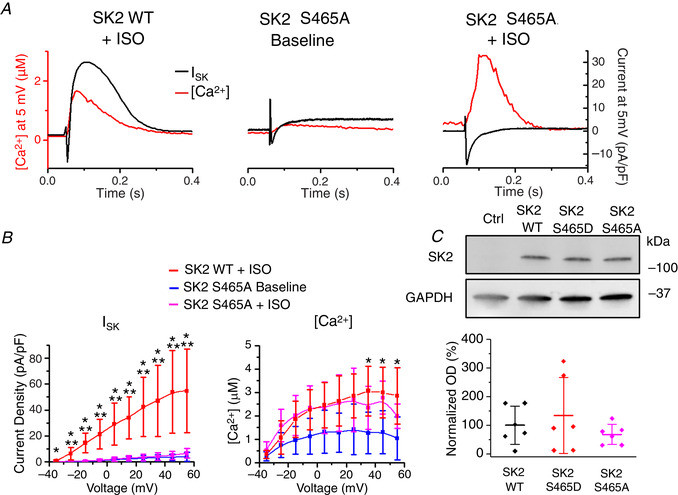
**Cultured rat VMs overexpressing SK2 phosphomimetic mutant S465A show no SK current** *A*, representative traces of [Ca^2+^]_i_ transients and *I*_SK_ at baseline and after incubation with ISO (100 nmol L^–1^, 3 min) in VMs expressing the rSK2‐S465A mutant or WT rSK2 (from Fig. 10) *B*, pooled mean ± SD *I*–*V* and peak [Ca^2+^]/*V* relationships for (A), *n* = 5–6, *N* = 5 and rSK2 WT + ISO data from Fig. 6. *I*_SK_: ^*^ *P* = 0.02 (−35 mV), *P* = 0.005 (−25 mV), *P* = 2.2 × 10^−4^ (−15 mV), *P* = 4 × 10^−4^ (−5 mV), *P* = 6.6 × 10^−4^ (5 mV), *P* = 1.8 × 10^−4^ (15 mV), *P* = 5.7 × 10^−4^ (25 mV), *P* = 0.001 (35 mV), *P* = 0.001 (45 mV), *P* = 0.001 (55 mV) WT ISO *vs*. S465A baseline. ^**^ *P* = 0.01 (−25 mV), *P* = 4.8 × 10^−4^ (−15 mV), *P* = 7.6 × 10^−4^ (−5 mV), *P* = 0.001 (5 mV), *P* = 3.9 × 10^−4^ (15 mV), *P* = 0.001 (25 mV), *P* = 0.002 (35 mV), *P* = 0.003 (45 mV), *P* = 0.004 (55 mV) rSK2 WT ISO *vs*. rSK2‐S465A ISO. [Ca^2+^]: ^*^ *P* = 0.02 (35 mV), *P* = 0.03 (45 mV), *P* = 0.002 (55 mV) WT ISO *vs*. S465A baseline, one‐way ANOVA with a Bonferroni *post hoc* test. ***C***, representative western blots from VMs expressing rSK2 WT, rSK2‐S465D and rSK2‐S465A.

**Figure 18:**
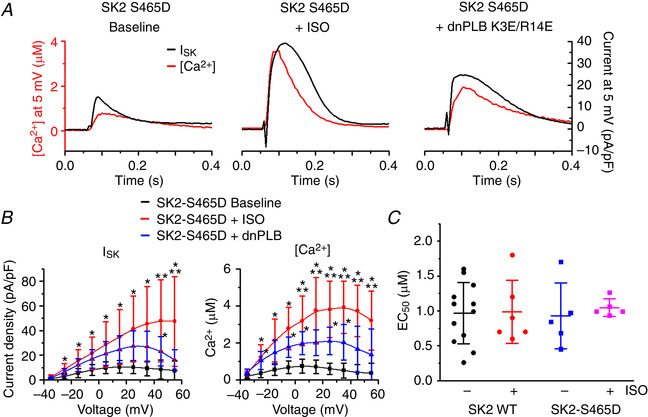
**SK2 phosphomimetic mutation S465D alleviates voltage‐dependent inhibition of SK channels by [Ca^2+^]** *A*, representative traces of [Ca^2+^]_i_ transients and *I*_SK_ at baseline and after incubation with ISO (100 nmol L^–1^ for 3 min) in VMs expressing the rSK2‐S465D mutant and dnPLB. *B*, pooled mean ± SD *I*–*V* and peak [Ca^2+^]/*V* relationships for (A), *n* = 7–8, *N* = 6. *I*_SK_: ^*^ *P* = 0.01 (−25 mV), *P* = 0.02 (−15 mV), *P* = 0.03 (−5 mV), *P* = 0.03 (5 mV), *P* = 0.02 (15 mV), *P* = 0.008 (25 mV), *P* = 0.006 (35 mV), *P* = 0.02 (45 mV, dnPLB), *P* = 0.008 (55 mV) *vs*. baseline. ^**^ *P* = 0.02 (45 mV), *P* = 0.03 (55 mV) *vs*. dnPLB. [Ca^2+^]: ^*^ *P* = 0.01 (−25 mV), *P* = 0.04 (−25 mV, dnPLB), *P* = 0.004 (−15 mV), *P* = 1.4 × 10^−4^ (−5 mV), *P* = 0.03 (−5 mV, dnPLB), *P* = 1.4 × 10^−4^ (5 mV), *P* = 0.04 (5 mV, dnPLB), *P* = 1.7 × 10^−4^ (15 mV), *P* = 2.2 × 10^−5^ (25 mV), *P* = 0.02 (25 mV, dnPLB), *P* = 1.5 × 10^−5^ (35 mV), *P* = 0.03 (35 mV, dnPLB), *P* = 6.4 × 10^−5^ (45 mV), *P* = 0.001 (55 mV) *vs*. baseline. ^**^ *P* = 0.047 (5 mV), *P* = 0.02 (15 mV), *P* = 0.01 (25 mV), *P* = 0.006 (35 mV), *P* = 0.006 (45 mV), *P* = 0.03 (55 mV) *vs*. dnPLB, one‐way ANOVA with a Bonferroni *post hoc* test. *C*, plot of EC_50_ values for rSK2 WT and the rSK2‐S465D recorded under basal conditions and in the presence of the β‐adrenergic agonist ISO (100 nmol L^–1^). Mean ± SD of data indicated by line. *P* = 1 for all comparisons, *n* = 5‐12, *N* = 4–5, one‐way ANOVA.

**Figure 19:**
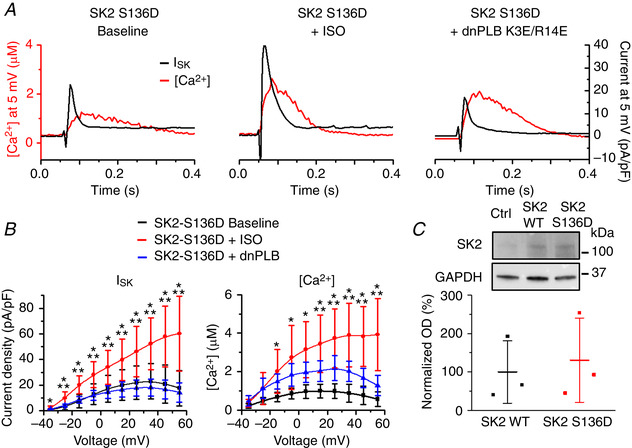
**N‐terminus S136 does not confer PKA‐dependent changes in SK2 function** *A*, representative traces of [Ca^2+^]_i_ transients and *I*_SK_ at baseline and after incubation with ISO (100 nmol L^–1^) in VMs expressing the rSK2‐S136D mutant and dnPLB. *B*, pooled mean ± SD *I*–*V* and peak [Ca^2+^]/*V* relationships for (A). *n* = 6–8, *N* = 5–6 per group. *I*_SK_: ^*^ *P* = 0.02 (−35 mV), *P* = 0.001 (−25 mV), *P* = 5.8 × 10^−4^ (−15 mV), *P* = 0.005 (−5 mV), *P* = 0.02 (5 mV), *P* = 0.02 (15 mV), *P* = 0.01 (25 mV), *P* = 0.004 (35 mV), *P* = 0.002 (45 mV), *P* = 0.001 (55 mV) *vs*. baseline. ^**^ *P* = 0.01 (−25 mV), *P* = 0.003 (−15 mV), *P* = 0.006 (−5 mV), *P* = 0.01 (5 mV), *P* = 0.01 (15 mV), *P* = 0.007 (25 mV), *P* = 0.002 (35 mV), *P* = 0.001 (45 mV), *P* = 0.001 (55 mV) *vs*. dnPLB. [Ca^2+^]: ^*^ *P* = 0.01 (−15 mV), *P* = 0.01 (−5 mV), *P* = 0.001 (5 mV), *P* = 5.1 × 10^−4^ (15 mV), *P* = 2.5 × 10^−4^ (25 mV), *P* = 1.7 × 10^−4^ (35 mV), *P* = 7.4 × 10^−5^ (45 mV), *P* = 1.5 × 10^−4^ (55 mV) *vs*. baseline. ^**^ *P* = 0.049 (15 mV), *P* = 0.04 (25 mV), *P* = 0.02 (35 mV), *P* = 0.004 (45 mV), *P* = 0.002 (55 mV) *vs*. dnPLB, one‐way ANOVA with a Bonferroni *post hoc* test. ***C***, representative western blots from VMs expressing rSK2 WT or rSK2‐S136D.

A phosphoproteomics study revealed that, out of >40 serine/threonine residues in rSK2, only five can be phosphorylated by PKA (Blom *et al*. 1999). Based on experiments in a heterologous system, Ren *et al*. (2006) proposed that phosphorylation of three adjacent C‐terminal serines (S568‐570) in HEK293 cells reduced the incorporation of channels into the surface membrane. These results are not easy to reconcile with the enhanced SK activity in VMs from diseased hearts. Also, it was shown that another SK isoform, SK3, is inhibited by cAMP‐PKA (Clarysse *et al*. 2014). The SK3 isoform encompasses two out of three homologous C‐terminus serines (S719‐720) and an N‐terminus serine‐285 homologous to rSK2 serine‐136 (S136). Sequence comparison shows that the remaining SK2 PKA phosphorylation site, serine‐465 (S465), is absent in rat SK3 (Fig. 16). Therefore, we focused on delineating the possible functional relevance of C‐terminus S465, which resides in CaM‐binding domain of SK2 (Li W *et al*. 2009; Ren *et al*. 2006; Schumacher *et al*. 2001). We produced an adenoviral vector carrying the phosphomimetic mutants rSK2‐S465A and rSK2‐S465D constructs for functional tests in cultured adult rat VMs (Figs 17 and 18). In addition, we generated another phosphomimetic mutant of rSK2, S136D, for comparison (Fig. 19). This N‐terminus serine is conserved between all three SK isoforms (Fig. 16). Similar to rSK2 WT overexpression studies (Fig. 14), experiments in rSK2‐S465D and rSK2‐S136D expressing VMs demonstrated ISO‐mediated relief of *I*_SK_ rectification paralleled by an increase in Ca^2+^ transient amplitudes (Figs 18 and 19, respectively). No SK current was observed in VMs expressing the dephosphomimetic mutant rSK2‐S465A (Fig. 17). To determine the direct effects of PKA‐mediated phosphorylation at S465 and S136 as opposed to secondary effects of ISO‐induced increased [Ca^2+^]_i_, we coexpressed rSK2 phosphomimetic mutants with dnPLB. The expression of dnPLB increases [Ca^2+^]_i_ in the absence of PKA‐mediated effects. Importantly, the current density was significantly increased in VMs coexpressing rSK2‐S465D and dnPLB but not rSK2‐S136D (Figs 18*B* and 19*B*, respectively). Moreover, in the presence of dnPLB, rectification of *I*_SK_ that occurs at higher voltages and [Ca^2+^] was significantly relieved only in the rSK2‐S465D group (Fig. 18*B*, blue *vs*. black line) as opposed to rSK2‐WT (Fig. 14) or rSK2‐S136D (Fig. 19), providing evidence that phosphorylation at this particular site underlies functional upregulation, reducing voltage‐dependent channel inhibition by high [Ca^2+^]_i_. Importantly, the sensitivity to [Ca^2+^] activation of rSK2 was not changed by S465D mutation (Fig. 18*C*).

**Figure 20:**
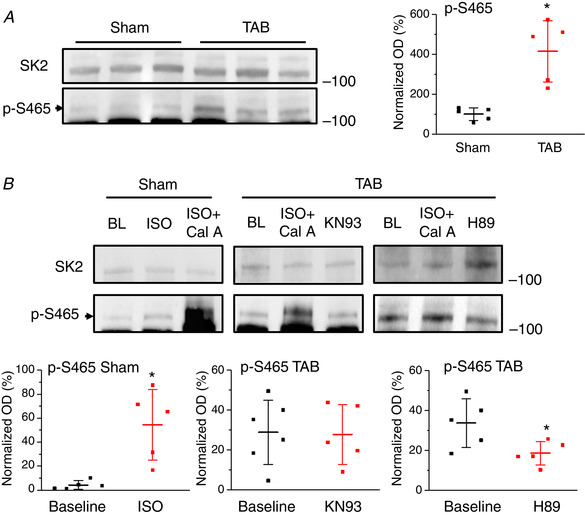
**Enhanced phosphorylation of SK2‐S465 in TAB rats VMs is PKA‐dependent** *A*, representative western blot of SK2 and phosphorylated S465 (left) and plot of p‐S465 band optical density (OD) normalized to SK2 levels for Sham and TAB rats (right). Each lane represents a sample from a separate rat. ^*^ *P* = 0.005, paired Student's *t* test. *B*, representative western blot of SK2 and phosphorylated S465 (top) and plots of p‐S465 band OD normalized to SK2 levels for Sham and TAB rats under baseline conditions, or treated with ISO (^*^ *P* = 0.01, paired Student's *t* test), KN93 or H89 (^*^ *P* = 0.04, one‐way ANOVA with a Bonferroni *post hoc* test) (bottom).

Next, we obtained custom antibodies that specifically recognize phospho‐S465. The western blot analysis presented in Fig. 20 confirmed that there is phosphorylation of this site in TAB VMs, which can be reduced in the presence of PKA inhibitor H89 (1 μmol L^–1^ for 30 min) but not CaMKII inhibitor KN93 (500 nmol L^–1^ for 30 min) (for comparison, see Fig. 3). Incubation of Sham VMs with ISO (100 nmol L^–1^ for 5 min) enhanced phosphorylation of S465.

## Discussion

SK channels are rapidly gaining recognition as a novel therapeutic target to treat cardiac arrhythmias (Chiamvimonvat *et al*. 2017; Clements *et al*. 2015; Heijman & Dobrev, 2017). However, their anti‐arrhythmic potential cannot be fully appreciated because of an incomplete understanding of mechanisms regulating SK channel function. Using a rat model of pressure‐overload‐induced hypertrophy, TAB, we aimed to delineate the molecular mechanisms underlying functional recruitment of SK channels in the VMs from diseased hearts. The results of the present study demonstrate that, under conditions of enhanced adrenergic drive such as hypertrophy, enhanced PKA phosphorylation of SK channels at a specific C‐terminus serine within the CaM‐binding domain (S465 for rat SK2) attenuates the inhibition of the channels that normally occurs at higher voltages and concentrations of intracellular Ca^2+^. The results provide the first evidence that, under β‐adrenergic stimulation, PKA phosphorylation of SK channels evokes *I*_SK_ that contributes to repolarization in healthy VMs and *ex vivo* hearts from Sham animals.

### PKA phosphorylation as a mechanism for functional recruitment of SK channels in ventricular myocytes from healthy and diseased hearts

It is well established that SK channels expressed in VMs are dormant in health but become functional in cardiac disease (Bonilla *et al*. 2014; Chang *et al*. 2013b; Chua *et al*. 2011; Lee *et al*. 2013; Mahida *et al*. 2014; Ni *et al*. 2013). The mechanism underlying this process remains unclear. Originally, it was considered that the enhanced activity of SK channels in cardiac disease could be explained by an increase in expression levels (Bonilla *et al*. 2014; Chang *et al*. 2013b; Ni *et al*. 2013). However, in our recent work, we found that the levels of SK channels in samples from the membrane fraction of isolated TAB VMs are significantly decreased compared to levels of SK channels in Sham VMs, eliminating such a possibility (Kim *et al*. 2017). Yang *et al*. (2015) proposed that decreased CK2 abundance leads to dephosphorylation of SK‐bound CaM at Thr79, thereby increasing the sensitivity of SK channels to activating [Ca^2+^]. Other studies attributed dephosphorylation of CaM to the increase of activity/levels of phosphatase PP2A tethered to the complex (Allen *et al*. 2007; Bildl *et al*. 2004). The results of western blot analysis of immunoprecipitated SK2 complexes (Fig. 2*D*) suggest that this is not the case for hypertrophic VMs from TAB rats because there are no changes in the phosphorylation of CaM and the levels of CK2 and the catalytic subunit of PP2A‐C in SK channel complex (Fig. 2*D* and *E*). Two recent reports implicate CaMKII‐mediated phosphorylation of the channel in this process (Mizukami *et al*. 2015; Tenma *et al*. 2018). Indeed, our experiments with phospho‐specific antibodies confirm that SK2 channels from TAB VMs are phosphorylated by serine/threonine kinases (Fig. 2 and 3) and both CaMKII and PKA are more active in myocytes from hypertrophic hearts compared to controls (Fig. 3*A*–*D*). However, SK2 phosphorylation was not reversed by the pharmacological CaMKII inhibitor KN93, in contrast to the PKA inhibitor H89, which was effective (Fig. 3*E* and *F*). Similar results were obtained in experiments using a specific anti‐phospho‐SK2‐S465 antibody (Fig. 20). The major role of PKA in modulation of SK activity as assessed in our functional studies is supported by the following findings: (i) specific PKA inhibitor PKI diminishes *I*_SK_ in VMs from TABs (Fig. 4); (ii) application of β‐adrenergic agonist ISO to activate PKA evokes *I*_SK_ in myocytes from Shams (Figs 7*A* and *B* and 8*A*), which prompts apparent contribution of SK channels to repolarization unmasked by APA both in VMs (Fig. 7*C*) and *ex vivo* optically mapped hearts from healthy animals (Fig. 9); (iii) a similarity of the effects of phosphomimetic mutation rSK2‐S465D and ISO on *I*_SK_ rectification in adult rat cultured VMs SK2‐overexpression experimental system (Figs 14 and 18); and (iv) alleviation of *I*_SK_ by introducing dephosphomimetic mutation S456A (Fig. 17). In line with our data, a recent report demonstrated an enhancement of *I*_SK_ in rabbit hearts challenged with ISO (Chen *et al*. 2018). Taken together, these results support the central role of PKA as a regulator of SK channel activity in cardiac hypertrophy. Furthermore, these data provide an insight into the role of SK channels in normal physiology as an integral part of the response to catecholaminergic surge during stress, providing additional repolarization to mitigate the depolarizing force of increased *I*_Ca_ and *I*_NCX_ to limit proarrhythmic incidences such as triggered activity.

Notably, previous studies using overexpressed SKs in heterologous experimental cell systems demonstrated PKA‐mediated functional downregulation of the channels (Clarysse *et al*. 2014; Ren *et al*. 2006), which contradicts our data obtained in native VMs. Ren *et al*. (2006) showed that activation of PKA in COS7 cells evoked almost complete translocation of SK2 from the surface membrane to the cytosol. Our recent report (Kim *et al*. 2017) confirms that, in TAB VMs, the levels of sarcolemmal SK2 and SK3 are lower than in Sham VMs. However, 20% downregulation of SK2 in VMs was not nearly as dramatic as the rapid and complete loss of plasmalemmal channels in COS7 cells. This suggests that strong association with other proteins such as LTCCs and α‐actinin (Lu *et al*. 2009; Zhang *et al*. 2017; Zhang *et al*. 2018) effectively interferes with retrograde transport of SK channels from the plasmalemma of VMs, which results in net functional upregulation of the channels by PKA phosphorylation. This may explain the well‐established presence of *I*_SK_ under conditions accompanied by enhanced catecholaminergic drive, such as hypertrophy or heart failure.

### Biphasic response of SK channels to [Ca^2+^]_i_ and its modulation by PKA in ventricular myocytes

Detailed knowledge of the control of SK channel gating by [Ca^2+^] has accumulated over the last 20 years of research. Submicromolar [Ca^2+^]_i_ effectively activates SK channels via constitutively bound CaM with an EC_50_ of ∼0.3–1 μmol L^–1^ (Adelman *et al*. 2012; Li N *et al*. 2009; Soh & Park, 2002; Tuteja *et al*. 2010; Xia *et al*. 1998). Because, in the majority of cell types, higher elevations of [Ca^2+^]_i_ are rare, the fact that SK channels can be inhibited by [Ca^2+^]_i_ in a voltage‐dependent manner with an IC_50_ of ∼20 μmol L^–1^ (Soh & Park, 2002) is often overlooked. The latter cannot be discounted in VMs where submembrane [Ca^2+^] has been estimated to reach 20 or even 100 μmol L^–1^ in the dyad during the peak of Ca^2+^ transient (Antoons *et al*. 2011; Cannell *et al*. 2013; Shannon *et al*. 2004). Recent work using super‐resolution microscopy places SK channels within a nanodomain also occupied by LTCCs and RyR2s in VMs (Zhang *et al*. 2018). The close proximity to these major sources of [Ca^2+^] further highlights the potential role of Ca^2+^‐dependent inhibition of SK channels during Ca^2+^ cycling. Our BN‐PAGE experiments confirm the physical interaction of SK2 with α1c subunit of LTCC (Fig. 10). Further immunolocalization studies using PLA showed that plasmalemmal SK2‐LTCC complexes are not precisely aligned with clusters of SR Ca^2+^ release channels RyR2s (Figs 11 and 12). The absence of SK channels in the dyad probably prevents ultrarapid exposure to very high dyadic [Ca^2+^] during Ca^2+^ release, which is estimated to be 100–200 μmol L^–1^ (Antoons *et al*. 2011; Cannell *et al*. 2013; Shannon *et al*. 2004) and is 5‐ to 10‐fold higher that the reported IC_50_ (20 μmol L^–1^; Soh & Park, 2002).

The presence of functional SK channels in diseased VMs usually is inferred from the effect of APA on APD, and currents are routinely measured before and after APA under conditions where [Ca^2+^]_i_ is clamped in the submicromolar range with Ca^2+^ chelators (Chang *et al*. 2013b; Gui *et al*. 2012; Yu *et al*. 2014; Zhang *et al*. 2014). By contrast, our cell electrophysiology experiments in conjunction with confocal Ca^2+^ imaging and analysis were specifically designed to assess SK function within the full physiological range of [Ca^2+^] in the vicinity of the channel. Outward *I*_SK_ (i.e. SK inhibitor‐sensitive component) measured in voltage clamped VMs from TABs (Figs 4 and 5) and Shams under ISO stimulation (Figs 7 and 8*A*) exhibited very fast activation kinetics followed by a rapid drop, presumably because [Ca^2+^]_i_ rising during the Ca^2+^ transient reaches concentrations sufficient to inhibit the channel. Analysis of dynamics of submembrane Ca^2+^ during the Ca^2+^ transient in voltage clamped TAB and Sham VMs at −10 mV revealed that, in the presence of ISO, *I*_SK_ reaches its maximum amplitude within an average of 10 ms (Fig. 13*B*) when [Ca^2+^]_sm_ is ∼10 μmol L^–1^ (Fig. 13*D*). A further increase in [Ca^2+^]_sm_, which lasts for additional 10–12 ms, evokes robust *I*_SK_ decay. Because of the dual action of Ca^2+^ (i.e. activation and inactivation), the dynamics of the *I*_SK_ response differs from the response of NCX1, another plasmamembrane Ca^2+^‐regulated transporter with similar EC_50_ ∼400 nmol L^–1^, which closely follows both rise and decay of [Ca^2+^]_sm_ (Weber *et al*. 2002).

Importantly, *I*_SK_ in native VMs was detectable only under conditions where PKA was active and it exhibited rectification with peak amplitude reached at –10 to −20 mV followed by a robust decrease at higher voltages (Figs 4*C*, 5, 7*B* and 8*A*). In cultured VMs overexpressing rSK2 channels, ISO completely alleviated rectification, whereas the *I*–*V* curve of *I*_SK_ under baseline showed a peak at ∼10 mV (Fig. 14). Nevertheless, freshly isolated VMs and the overexpression system both produced qualitatively similar results, implying that the main effect of PKA phosphorylation is to reduce the Ca^2+^/voltage‐dependent inhibition of the channels. Indeed, our experiments using intrinsic Ca^2+^ handling machinery to produce a ramp of [Ca^2+^]_i_ to assess the sensitivity of SK channels within its activation range demonstrated no change in EC_50_ ∼1 μmol L^–1^ before and after challenge with ISO (Fig. 15). This is in contrast to previous findings in VMs from failing hearts where enhanced sensitivity to activation by Ca^2+^ was proposed to underlie disease‐associated upregulation of SK channels (Bildl *et al*. 2004; Chang *et al*. 2013b; Chua *et al*. 2011; Ni *et al*. 2013; Yang *et al*. 2015). Most probably, this discrepancy stems from an underestimation of the role of the Ca^2+^/voltage‐dependent channel inhibition as a result of challenges in the assessment of *I*_Max_ in the previous studies. Future detailed experimental studies in conjunction with spatially resolved computational modelling are needed to help settle this controversy.

Notably, the abundant divalent cation Mg^2+^ was also reported to inhibit SK channels in heterologous system (Soh & Park, 2001; Soh & Park, 2002). However, this inhibition was largely relieved in the presence of [Ca^2+^] at 2–20 μmol L^–1^ (Soh & Park, 2001) when [Mg^2+^] was close to its physiological range which is ∼1 mmol L^–1^ in VMs (Bers, 2002). Furthermore, the inhibitory action of Mg^2+^ may be substantially reduced in the presence of physiological levels of ATP, as the case of RyR2 (Bers, 2002). The free [Mg^2+^] in our pipette solution for cell electrophysiology experiments was 1.37 mmol L^–1^ (Maxchelator; Bers *et al*. 2010). Increasing it to supra‐physiological 5 mmol L^–1^ did not produce apparent effects on *I*_SK_ parameters in VMs overexpressing rSK2 (data not shown), suggesting that the role of [Mg^2+^]_i_ in dynamic regulation of SK channels in cardiac myocytes is relatively small compared to [Ca^2+^]_i_. Unfortunately, we were unable to assess sensitivity of SK channels to inhibition by Ca^2+^ in native Sham rat VMs with preserved Ca^2+^ cycling in the absence of PKA activation. Under these conditions, we were unable to record discernible *I*_SK_ despite of sufficient [Ca^2+^]_sm_ for activation of the channels. Future single channel patch clamp studies from native VMs using an inside–out configuration are warranted to address this question.

Functional upregulation of SK channels can be viewed as an adaptive mechanism to reduce risk of Ca^2+^‐dependent arrhythmia in diseased hearts (Bonilla *et al*. 2014; Chang *et al*. 2013b; Clements *et al*. 2015; Kim *et al*. 2017). It is clear that this protection is limited because arrhythmia still persists under conditions such as heart failure. It is tempting to hypothesize that, if indeed SK channel sensitivity to activating [Ca^2+^] is not changed under disease‐related remodelling, there is a reasonable rationale for using pharmacological agents that act by enhancing SK Ca^2+^ sensitivity (Hougaard *et al*. 2004; Kim *et al*. 2017; Strobaek *et al*. 2004). This could potentially further facilitate repolarization and thereby reduce the risk of Ca^2+^‐dependent ventricular tachyarrhythmias.

In conclusion, the results of the present study show that the functional upregulation of SK2 channels in hypertrophic rat ventricular cardiomyocytes is driven by enhanced PKA‐dependent phosphorylation at S465. PKA phosphorylation attenuates *I*_SK_ rectification by reducing the Ca^2+^/voltage‐dependent inhibition of SK channels without changing their sensitivity to activating submicromolar [Ca^2+^]. This mechanism underlies the functional recruitment of SK channels not only in cardiac disease, but also in normal physiology, contributing to repolarization under conditions of enhanced adrenergic drive.

## Additional information

### Competing interests

The authors declare that they have no competing interests.

### Author contributions

DT, BRC and GK participated in the study design. SH and DT wrote first draft of manuscript. SH, IP, RT, PB, TYK, KR and RTC performed the experiments. SH, IP, RT, PB, TYK, KR, RTC, BRC and DT conducted data interpretation and analyses. SH, IP, RT, PB, TYK, KR, RTC, BRC, GK and DT reviewed the manuscript submitted for publication. All authors revised and approved the final version of the manuscript. All persons designated as authors qualify for authorship, and all those who qualify for authorship are listed.

### Funding

This work was supported by American Heart Association Grant #18POST33960456 (SH); National Heart, Lung, and Blood Institute at the National Institutes of Health (NIH) NIH RO1HL135236 (RTC); NIH R01HL110791 (GK); NIH R01HL096669 (BRC); and American Heart Association Grant in Aid 15GRNT25650002 and NIH R01HL121796 (DT).
